# Phase Modulation for Quadruplex Channels of Arbitrary Orthogonal Polarization States via Bilayer Metasurfaces

**DOI:** 10.1002/nap2.70012

**Published:** 2026-01-16

**Authors:** Wei Wang, Jun Wang, Qiaohua Wu, Jie Lin, Peng Jin, Shutian Liu, Zhongyi Guo, Keya Zhou

**Affiliations:** ^1^ School of Physics Harbin Institute of Technology Harbin China; ^2^ Key Laboratory of Micro‐Systems and Micro‐Structures Manufacturing Harbin Institute of Technology Ministry of Education Harbin China; ^3^ School of Instrumentation Science and Engineering Harbin Institute of Technology Harbin China; ^4^ School of Computer and Information Hefei University of Technology Hefei China

**Keywords:** bilayer metasurfaces, holography, multi‐channel multiplexing, orbital angular momentum, orthogonal states of polarization

## Abstract

Optical metasurfaces are widely studied due to their unprecedented wavefront modulation capabilities for multiple polarization channels. Current studies predominantly focus on complete polarization conversion. Recent progress indicates that the phases of quadruplex polarization channels can be independently modulated under incomplete polarization conversion conditions. However, these four‐channel phase modulation operations are limited to circular or linear polarization states and neglect amplitude modulation. Here, a strategy is proposed to achieve four‐channel phase modulation and flexible energy distribution of arbitrary orthogonal polarization states under incomplete polarization conversion conditions. Wavefront modulations for quadruplex channels of arbitrary orthogonal polarization states (circular, linear, elliptical, and first‐order cylindrically vectorial), such as orbital angular momentum manipulation, Bessel beam generation, deflection, and holography, are numerically demonstrated based on this strategy. Furthermore, the energy distribution of the quadruplex polarization channels is achieved by varying the polarization conversion efficiency. These operations are implemented through all‐dielectric free‐standing bilayer metasurfaces. The proposed design strategy extends the application of metasurfaces in multichannel optical field modulation.

## Graphical abstract

This work demonstrates a strategy for four‐channel phase modulation and flexible energy distribution of arbitrary orthogonal states of polarization under incomplete polarization conversion conditions. Such a strategy can be implemented through an all‐dielectric free‐standing bilayer metasurface configuration and can be used for multichannel optical field manipulation.

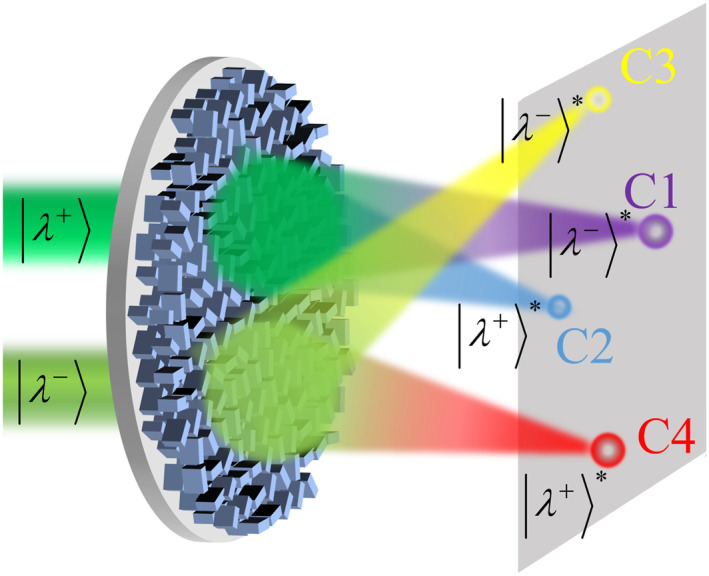

## 1 Introduction

Metasurfaces composed of subwavelength structures can be used to manipulate the properties of electromagnetic waves such as amplitude, phase, polarization, and frequency [1, 2, 3, 4, 5]. Because of their advantages of compactness and versatility, metasurfaces have been applied for various functions, including imaging [6, 7, 8], image processing [9, 10], holography [11, 12], integrated photonics [13, 14], and nonlinear optics [15, 16]. Initially, the geometric (Pancharatnam–Berry, PB) phase was used to manipulate the phase profiles of circularly polarized light [17, 18]. Nevertheless, phase decoupling of orthogonally circularly polarized beams cannot be achieved solely through the geometric phase. For example, a converging metalens based on geometric phase with design polarization of left‐handed circular polarization (LCP) becomes a diverging metalens when right‐handed circularly polarized (RCP) light is incident [17]. A method combining geometric and propagation phases has been utilized to realize decoupling of the phases of arbitrary orthogonal states of polarization [19]. This strategy has later been extended to decouple the amplitudes and the complex amplitudes of arbitrary orthogonal states of polarization [20, 21]. Nevertheless, these studies have concentrated on complete polarization conversion; that is, only two polarization channels can be modulated independently.

To further increase the number of functional channels, the supercells consisting of multiple nanopillars have been proposed for designing metasurfaces [22, 23, 24]. However, this design strategy results in a reduction of the spatial sampling rate. Metasurfaces consisting of the periodic arrangement of single nanopillars are capable of phase modulation of three circularly or linearly polarized channels under incomplete polarization conversion conditions [25, 26, 27, 28, 29]. Recently, these methods have been generalized to achieve complex amplitude manipulation for three polarization channels of arbitrary orthogonal states of polarization ($|\lambda^{+}\rangle$ and $|\lambda^{-}\rangle$) [30]. Notably, there are four polarization channels under incomplete polarization conversion conditions, including two complex conjugate states (${|\lambda^{+}\rangle }^{*}$ and ${|\lambda^{-}\rangle }^{*}$) and two orthogonal complex conjugate states (${|\lambda^{-}\rangle }^{*}$ and ${|\lambda^{+}\rangle }^{*}$). In the previous work, only three polarization channels are modulated independently, and two polarization channels (two orthogonal complex conjugate states ${|\lambda^{-}\rangle }^{*}$ and ${|\lambda^{+}\rangle }^{*}$) have identical responses [25, 26, 27, 28, 29, 30]. Single‐layer metasurfaces designed based on spatial multiplexing methods, as well as multilayer and cascaded metasurfaces, have been used for phase manipulation of quadruplex polarization channels [31, 32, 33, 34, 35]. Nevertheless, these studies on modulating quadruplex polarization channels focus on orthogonal circular or linear polarization states. In addition, the energy distribution of different polarization channels is neglected. These defects hinder the further application of metasurfaces in polarization optics.

In this work, we propose a strategy for achieving amplitude and phase modulation for four channels of arbitrary orthogonal polarization under incomplete polarization conversion conditions. Based on this strategy and bilayer metasurfaces, we demonstrate the generation of vortex and Bessel beams, deflection, holography, and focusing within the quadruplex channels of orthogonal circular, linear, elliptical, and cylindrical vectorial polarization states. Moreover, the energy distribution of the quadruplex elliptical polarization channels is achieved by varying the polarization conversion efficiency. The proposed strategy improves the phase modulation method for quadruplex polarization channels under partial polarization conversion conditions, which will further extend the application of metasurfaces in the field of multichannel optical field modulation.

## 2 Results and Discussion

### 2.1 Principle and Meta‐Atom

**FIGURE 1:**
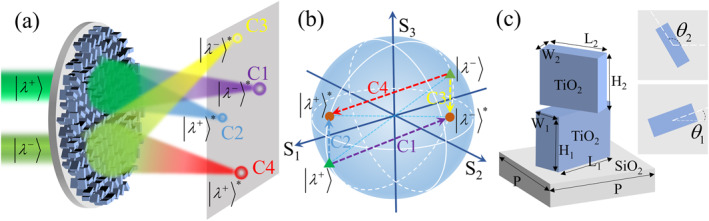
Free‐standing bilayer metasurface and the configuration of the meta‐atom. (a) Functional diagram of the designed free‐standing bilayer metasurface. The bilayer metasurface controls the phases and energies of the quadruplex polarization channels under the condition of incomplete polarization conversion. Here, the four channels are notated as C1 (from $|\lambda^{+}\rangle$ to ${|\lambda^{-}\rangle }^{*}$), C2 (from $|\lambda^{+}\rangle$ to ${|\lambda^{+}\rangle }^{*}$), C3 (from $|\lambda^{-}\rangle$ to ${|\lambda^{-}\rangle }^{*}$), and C4 (from $|\lambda^{-}\rangle$ to ${|\lambda^{+}\rangle }^{*}$). (b) The positions of the input and output polarization states on the Poincaré sphere. (c) Configuration diagram of the free‐standing bilayer meta‐atom. The meta‐atom consists of a SiO_2_ substrate and two vertically stacked TiO_2_ nanopillars surrounded by air. The period of the meta‐atom is 360 nm, and the heights of the bottom and top nanopillars are 650 nm. The bottom nanopillars in this paper are equivalent to half‐wave plates (HWPs).

The schematic diagram of the four‐channel modulation of arbitrary orthogonal polarization states through a free‐standing bilayer metasurface is shown in Figure 1a. Because of incomplete polarization conversion, any orthogonal states of polarization ($|\lambda^{+}\rangle$ and $|\lambda^{-}\rangle$) are converted to orthogonal complex conjugate states (${|\lambda^{-}\rangle }^{*}$ and ${|\lambda^{+}\rangle }^{*}$) and complex conjugate states (${|\lambda^{+}\rangle }^{*}$ and ${|\lambda^{-}\rangle }^{*}$). The positions of the input (green triangles) and output (brown circles) polarization states on the Poincaré sphere are illustrated in Figure 1b. The four polarization conversion channels are noted as C1 (from $|\lambda^{+}\rangle$ to ${|\lambda^{-}\rangle }^{*}$), C2 (from $|\lambda^{+}\rangle$ to ${|\lambda^{+}\rangle }^{*}$), C3 (from $|\lambda^{-}\rangle$ to ${|\lambda^{-}\rangle }^{*}$), and C4 (from $|\lambda^{-}\rangle$ to ${|\lambda^{+}\rangle }^{*}$), respectively, and are indicated by arrows of different colors in Figure 1b. The phase profiles and energy ratios of the quadruplex polarization channels are manipulable, typically, as illustrated in Figure 1a, in which the focus positions and intensities of the four polarization channels are different.

The meta‐atom composing the free‐standing bilayer metasurface is shown in Figure 1c, which consists of a silica (SiO_2_) substrate and two stacked titanium dioxide (TiO_2_) nanopillars surrounded by air. This configuration has been proven to be manufacturable [36], and it enhances refractive index contrast while evading unwanted reflections and optical losses induced by interlayer gaps [36, 37, 38, 39]. An independent nanopillar can usually be described by the Jones matrix [19]:

(1) $$M=R\left(-\theta \right)\left[\begin{array}{cc} e^{i\varphi_{x}} & 0\\ 0 & e^{i\varphi_{y}} \end{array}\right]R\left(\theta \right)$$

where $\varphi_{x}$ and $\varphi_{y}$ are the phase delays of the linearly polarized light along the *x* and *y* directions, respectively, and R(θ) is the rotation matrix. Neglecting the coupling effect [40, 41], the optical response of vertically stacked dual nanopillars can be expressed as $M=M_{2}\cdot M_{1}$, where Subscripts 1 and 2 denote the bottom and top nanopillars, respectively.

Under the condition of incomplete polarization conversion, the modulation of the orthogonally polarized beams by the bilayer metasurface is expressed as follows [30]:

(2) $$\left\{\begin{array}{l} M|\lambda^{+}\rangle =f_{1}{|\lambda^{-}\rangle }^{*}+f_{2}{|\lambda^{+}\rangle }^{*}\\ M|\lambda^{-}\rangle =f_{3}{|\lambda^{-}\rangle }^{*}+f_{4}{|\lambda^{+}\rangle }^{*} \end{array}\right.$$

where *f* corresponds to the complex amplitude distributions of the quadruplex polarization channels. Previous studies have demonstrated that single‐layer metasurfaces can modulate the phase distributions of the four polarization channels [27, 30], but the C1 and C4 channels have the same response ($f_{1}=f_{4}$). In other words, only three polarization channels can be independently controlled. To provide additional control parameters for manipulating the phases of quadruplex channels with arbitrary orthogonal polarization states, the bottom nanopillars of the free‐standing bilayer meta‐atoms are configured as HWPs ($\varphi_{x1}-\varphi_{y1}=\pi$). A detailed analysis is provided in Note S1 of Supporting Information S1. Typically, when the bilayer metasurface is illuminated by orthogonally circularly polarized beams, the complex amplitude distributions of the four polarization channels are given as follows:

(3) $$\left\{\begin{array}{l} f_{1}=\sin\;\Delta e^{i\left(\varphi_{x1}+\sum +\pi /2+2\theta_{1}-2\theta_{2}\right)}\\ f_{2}=\cos\;\Delta e^{i\left(\varphi_{x1}+\sum +2\theta_{1}\right)}\\ f_{3}=\cos\;\Delta e^{i\left(\varphi_{x1}+\sum -2\theta_{1}\right)}\\ f_{4}=\sin\;\Delta e^{i\left(\varphi_{x1}+\sum +\pi /2-2\theta_{1}+2\theta_{2}\right)} \end{array}\right.$$

where $\Delta =\left(\varphi_{x2}-\varphi_{y2}\right)/2$ and $\sum =\left(\varphi_{x2}+\varphi_{y2}\right)/2$. Obviously, the quadruplex polarization channels have different phase distributions, and the intensity ratio of C1 and C2 (or C3 and C4) can be controlled by the phase difference Δ of the top nanopillar. Notice that the phases of the quadruplex channels are determined by four free parameters ($\varphi_{x1}$, ∑, $\theta_{1}$, and $\theta_{2}$), but these four parameters do not completely decouple the phases of the quadruplex channels. When the phases of any three channels are determined, the phase of the fourth channel will be fixed as well. In this case, the phase modulation of the quadruplex channels can be realized by obtaining approximate solutions with small deviations [30, 42].

### 2.2 Metasurfaces for Phase Modulation of Quadruplex Channels

**FIGURE 2:**
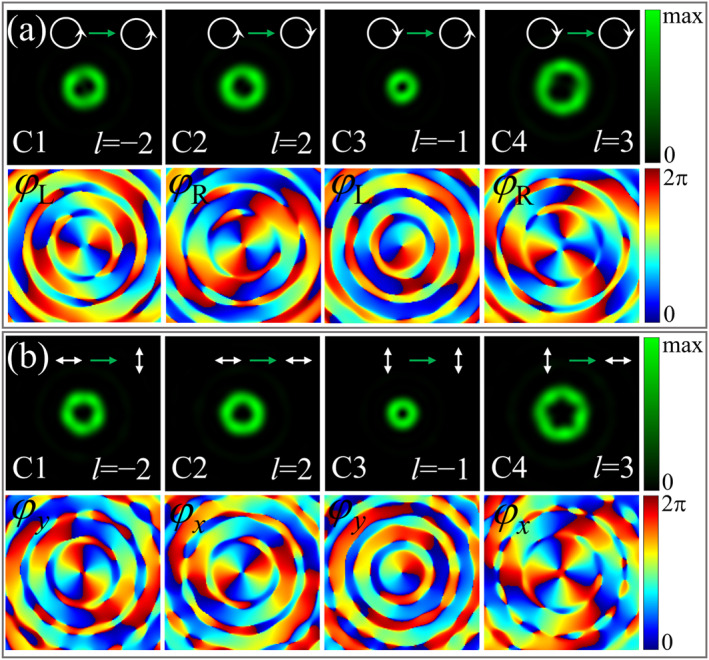
Intensity (top row) and phase (bottom row) distributions of electric field components of quadruplex channels at the design focal length (z=40μm) when orthogonally circularly (a) and linearly polarized (b) beams illuminate M1 and M2. The designed topological charges l of the vortex light for the four channels are −2, 2, −1, and 3, respectively.

At first, we demonstrate that the proposed design strategy is compatible with previously reported circular (or linear) polarization four‐channel phase modulation methods. We designed two free‐standing bilayer metasurfaces (noted as M1 and M2) to modulate the topological charges l of the circularly and linearly polarized vortex light, respectively. The operating wavelength of the designed metasurfaces is 532 nm, and the parameters of the meta‐atoms and acquisition method are given in Note S2 of Supporting Information S1. The topological charges l of the circular and linear polarization quadruplex channels are −2, 2, −1, and 3, respectively. The energies of the quadruplex polarization channels were set to be identical. Note S3.1 (Supporting Information S1) provides the detailed design approach. We simulated the metasurfaces using the finite‐difference time‐domain (FDTD) method. The top rows of Figure 2a,b, respectively, show the focal plane intensity distributions for the four polarization conversion channels of the two metasurfaces. The arrows at the top of each figure indicate the polarization conversion process, whereas the equations at the bottom (e.g., l=2) specify the designed topological charge. Corresponding phase distributions of the four channels are illustrated in the bottom rows of Figure 2a,b, where labels $\varphi_{L}$, $\varphi_{R}$, $\varphi_{y}$, and $\varphi_{x}$ in the upper left represent the phases of the output LCP, RCP, *y*‐polarized, and *x*‐polarized light, respectively. The phase distributions indicate that the topological charges l of the vortex light within the quadruplex channels are consistent with the design.

**FIGURE 3:**
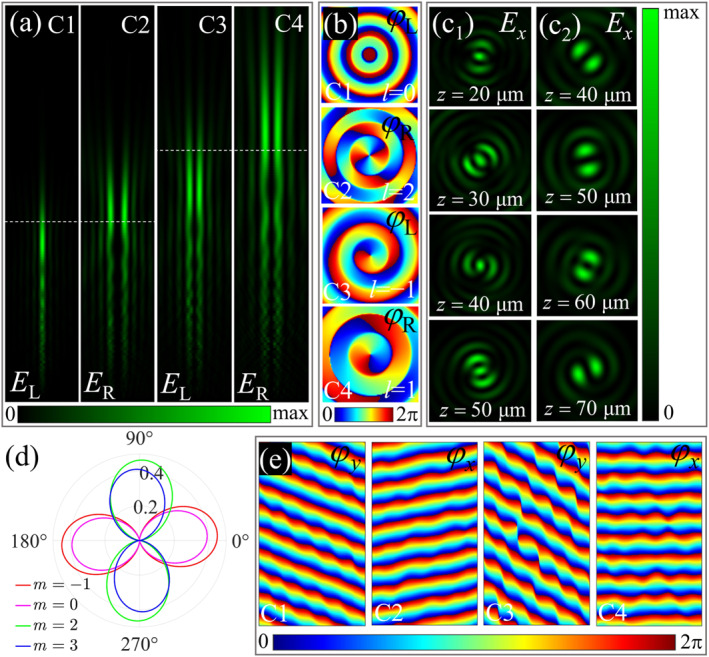
Bessel beams with longitudinally varying polarization states (M3) and the beams deflected within four channels (M4). (a) Longitudinal intensity distributions of the circularly polarized electric field components corresponding to the four circularly polarized channels of M3. (b) Transverse phase distributions of the circularly polarized electric field components at longitudinal positions of 50 and 70 μm (white dotted lines in (a)) within the four channels. (c) The intensity distributions of *x*‐polarized electric field components at different longitudinal positions when left‐ (C_1_) and right‐handed (C_2_) circularly polarized beams are incident. (d) Polarization state diagrams of the four designed diffraction orders *m* (*m* = 2, −1, 3, and 0) for M4. (e) Longitudinal phase distributions of *x*‐ and *y*‐polarized waves when orthogonally linearly polarized beams illuminate M4.

Further, we design a metasurface (M3) that generates vector Bessel beams with longitudinally varying polarization states under illumination with orthogonally circularly polarized beams. Vector Bessel beams with polarization states varying along the propagation direction can be generated by superimposing orthogonally circularly polarized Bessel beams with different longitudinal wave vectors and topological charges [43, 44]. The longitudinal wave vector (depth of focus) of the Bessel beam can be controlled by the numerical aperture (NA) of the metasurface [45]. Here, we set the numerical apertures of the quadruplex channels to 0.3, 0.25, 0.22, and 0.17, and the circularly polarized components carry topological charges of 0, 2, −1, and 1, respectively. The detailed design are provided in Note S3.2 (Supporting Information S1). The longitudinal distributions of the electric field component intensities of the four polarization channels obtained from the simulation are presented in Figure 3a. Obviously, the depths of focus (longitudinal wave vectors) of the circularly polarized Bessel beams in the quadruplex channels are different. Figure 3b demonstrates the transverse phase distributions of the orthogonally circularly polarized components of the electric field in the quadruplex channels at longitudinal positions of z=50μm and z=70μm (corresponding to the white dotted lines in Figure 3a). The transverse phase distributions indicate that the topological charges carried by the left‐ and right‐handed circular polarization components match the designed values. Figure 3c1,c2 shows the transverse intensity distributions of *x*‐polarized electric field components at different longitudinal positions when left‐ and right‐handed circularly polarized beams are incident, respectively. The varying orientations of the intensity lobes indicate that the polarization distributions of the Bessel beams generated by the superposition vary along the longitudinal direction [44]. See Note S3.2 of Supporting Information S1 for more analysis.

Additionally, we demonstrate a gradient metasurface (M4) engineered to realize deflections of orthogonally linearly polarized beams within four channels. M4 transmits the *x*‐ and *y*‐polarized beams in the four channels to the 2, −1, 3, and 0 diffraction orders, respectively. The supercell that makes up M4 is formed by eight meta‐atoms aligned along the *x* direction. The detailed design are presented in Note S3.3 (Supporting Information S1). The polarization state diagrams of the four target diffraction orders are shown in Figure 3d. The simulated degrees of linear polarization of the target polarization states within the 2, −1, 3, and 0 diffraction orders are 98.35%, 99.57%, 98.99%, and 98.10%, respectively. Figure 3e illustrates the longitudinal phase distributions of the transmitted *x*‐ and *y*‐polarized beams.

**FIGURE 4:**
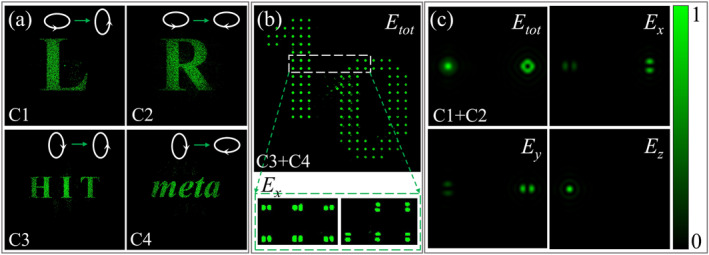
Holographic images encoded via elliptical (M5) and vectorial (M6) polarization states. (a) Four holographic images of Metasurface M5 encoded into orthogonal elliptical polarization $\left({\left[\begin{array}{cc} \sqrt{3}/2 & i/2 \end{array}\right]}^{T}\right.$ and $\left.{\left[\begin{array}{cc} 1/2 & -\sqrt{3}i/2 \end{array}\right]}^{T}\right)$ quadruplex channels. The C1, C2, C3, and C4 polarization channels correspond to the four strings “L,” “R,” “HIT,” and “meta,” respectively. (b) Holographic image generated by Metasurface M6 when azimuthally polarized light is incident. The inset shows the intensity distribution of the *x*‐polarized component of the electric field. Numbers 1 and 0 are encoded into radial and azimuthal polarization, respectively. (c) Intensity distributions of the total and components of the electric field of the separated tightly focused radially and azimuthally polarized beams generated by Metasurface M6 when illuminated by a radially polarized beam.

Elliptical polarization is the most common spatially homogeneous polarization. Next, we show independent phase control of orthogonal elliptical polarization quadruplex channels. We design a metasurface (M5) for encoding four independent holographic images (four strings “L,” “R,” “HIT,” and “meta”) on orthogonal elliptical polarization $\left({\left[\begin{array}{cc} \sqrt{3}/2 & i/2 \end{array}\right]}^{T}\right.$ and $\left.{\left[\begin{array}{cc} 1/2 & -\sqrt{3}i/2 \end{array}\right]}^{T}\right)$ quadruplex channels. The detailed design can be seen in Note S3.4 of Supporting Information S1. The holographic images of the four channels of M5 obtained from the simulation are displayed in Figure 4a. Obviously, the simulation results are almost identical to the design images, which means that the proposed design strategy applies to orthogonally elliptically polarized beams. Vector optical fields with inhomogeneous polarization distributions have been widely applied (e.g., multiplexing communications [46], super‐resolution imaging [47], particle manipulation [48], materials processing [49], and holography [50]), but in contrast to scalar optical fields, they have rarely been investigated as excitation sources in metasurface polarization optics. As a further validation of the proposed strategy, here we show a metasurface (M6) capable of modulating the phase distributions of the quadruple channels of first‐order cylindrical vectorial polarization (radial and azimuthal polarization). Specifically, M6 generates tightly focused radially and azimuthally polarized light when illuminated by radially polarized light. Additionally, M6 will produce two dot matrix holographic images (Numbers 1 and 0) encoded in radial and azimuthal polarization, respectively, when the incident light is switched to azimuthal polarization. See Note S3.5 (Supporting Information S1) for details. Figure 4b,c shows the simulation results when azimuthally and radially polarized plane waves illuminate M6, respectively. According to the holographic images and the inset of electric field component intensity shown in Figure 4b, Numbers 1 and 0 are encoded into radial and azimuthal polarization, respectively, which is consistent with the design. The intensity distributions of the electric field components demonstrated in Figure 4c indicate that the focused azimuthally polarized beam is purely transverse, whereas the longitudinal component intensity of the tightly focused radially polarized beam can significantly exceed the intensity of the transverse component, which is in agreement with the reported literature [51, 52].

### 2.3 Metasurfaces for Energy Distribution of Quadruplex Channels

**FIGURE 5:**
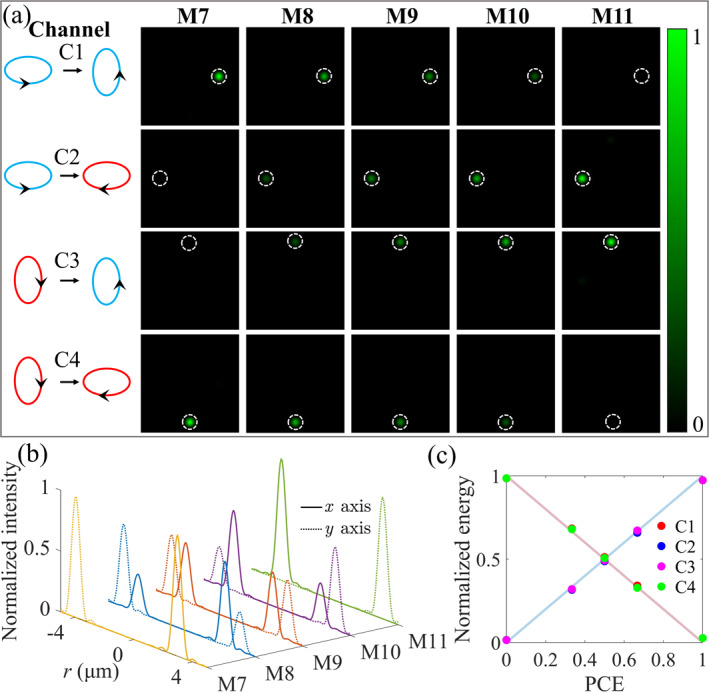
Energy ratios of elliptical polarization $\left({\left[\begin{array}{cc} \sqrt{3}/2 & i/2 \end{array}\right]}^{T}\right.$ and $\left.{\left[\begin{array}{cc} 1/2 & -\sqrt{3}i/2 \end{array}\right]}^{T}\right)$ four channels are manipulated based on the proposed strategy. (a) Intensity distributions of the focal planes of the four polarization channels for different polarization conversion efficiencies. The energy ratios of the C1 and C2 channels (or C4 and C3 channels) of the five metasurfaces are 1:0, 2:1, 1:1, 1:2, and 0:1, respectively. (b) Intensity distributions along the *x*‐axis (solid lines) and *y*‐axis (dashed lines) at the focal plane for the five metasurfaces (M7, M8, M9, M10, and M11). (c) Energy proportions of the four polarization channels for different PCEs.

Finally, we modulate the energy distribution of the quadruplex channels based on the proposed design strategy. The orthogonal states of elliptical polarization $\left({\left[\begin{array}{cc} \sqrt{3}/2 & i/2 \end{array}\right]}^{T}\right.$ and $\left.{\left[\begin{array}{cc} 1/2 & -\sqrt{3}i/2 \end{array}\right]}^{T}\right)$ are set as the modulation target without loss of generality. Five metasurfaces (noted as M7, M8, M9, M10, and M11, respectively) were designed, and they all enable the focusing of the optical field within the quadruplex channels to different locations in the same transverse plane, as shown in Figure 1a. The detailed information is provided in Note S3.6 of Supporting Information S1. The energy ratios of the C1 and C2 channels (or C4 and C3 channels) of the five metasurfaces are designed as 1:0, 2:1, 1:1, 1:2, and 0:1, respectively. The transverse intensity distributions of the electric field components of the four channels for the five metasurfaces are depicted in Figure 5a. It is obvious that as the polarization conversion efficiency (PCE) increases, the intensities of the C1 and C4 channels decrease, whereas the intensities of the C2 and C3 channels increase. Here, polarization conversion efficiency is defined as the ratio at which incident light is converted to its complex conjugate state. The focal plane intensity distributions along the *x*‐axis (solid lines) and *y*‐axis (dotted lines) are shown in Figure 5b. The energy evolution of the quadruplex polarization channels is displayed in Figure 5c. The accuracy, linearity, loss, and range of energy regulation are discussed in Note S3.6 of Supporting Information S1. These results match well with the design. Our results show that the proposed design strategy is capable of phase manipulation and energy distribution of arbitrary orthogonal polarization quadruplex channels. This phase modulation capability remains effective even under complete polarization conversion conditions, as evidenced by M11. We analyze the broadband response of the designed metasurfaces, taking M9 as a representative. The results show that the relative focusing efficiencies are greater than 80% within the 80‐nm range. Here, the relative focusing efficiency is defined as the ratio of the focusing efficiency at other wavelengths to that at the design wavelength. The detailed information is presented in Note S4 of Supporting Information S1.

Moreover, as demonstrated in Supporting Information S1: Note S5, the designed bilayer metasurface maintains robust performance under misalignment tolerance within 20 nm and structural dimensional error within 5 nm. The core of this paper lies in proposing a metasurface design strategy for manipulating the phase and energy of four arbitrary orthogonal polarization channels under the condition of incomplete polarization conversion. Note S6 of Supporting Information S1 demonstrates that the proposed four‐channel manipulation strategy can be implemented through the stacked metasurface configuration in addition to the free‐standing bilayer metasurface configuration. The configuration diagram of the meta‐atom constituting the stacked metasurface is depicted in Supporting Information S1: Figure S19, where the bottom nanopillar is embedded in a cladding [36]. Compared with the free‐standing bilayer metasurface configuration, the stacked metasurfaces have attracted more attention, and their manufacturing processes are more mature [37, 41, 53, 54].

## 3 Conclusion

In summary, this paper proposes a strategy for phase modulation and energy distribution of arbitrary orthogonal polarization quadruplex channels under incomplete polarization conversion conditions. Based on this strategy, four‐channel phase modulation for orthogonal circular, linear, elliptical, and cylindrical vectorial polarization states has been demonstrated, encompassing focusing, orbital angular momentum control, Bessel beam generation, deflection, and holography. In addition, the energy ratios of the four polarization channels can be controlled by the polarization conversion efficiency. The proposed strategy also enables dual‐channel phase manipulation of arbitrary orthogonal states of polarization under complete polarization conversion conditions. In addition to the free‐standing bilayer metasurface configuration, the phase manipulation strategy can also be implemented by the stacked metasurface configuration. Our work provides a practical pathway toward multichannel and multifunctional metasurfaces. The proposed strategy is expected to be extended to other frequency bands and find applications in orbital angular momentum multiplexing, holography, and beyond.

## Author Contributions

**Wei Wang:** conceptualization, visualization, formal analysis, writing – original draft, validation, methodology. **Jun Wang:** data curation, writing – review and editing. **Qiaohua Wu:** writing – review and editing, formal analysis. **Jie Lin:** conceptualization, methodology, project administration. **Peng Jin:** conceptualization, project administration. **Shutian Liu:** conceptualization, project administration. **Zhongyi Guo:** conceptualization, supervision. **Keya Zhou:** conceptualization, software, supervision, writing – review and editing, project administration, resources, funding acquisition.

## Funding

This work was supported by the National Key Research and Development Program of China (2017YFF0107502, 2018YFE0204000) and the National Natural Science Foundation of China (11874132, 12074094).

## Supporting information


Supporting Information S1

## Data Availability

The data that support the findings of this study are available from the corresponding author upon reasonable request.
