# Supplementary material for: Phase Modulation for Quadruplex Channels of Arbitrary Orthogonal Polarization States via Bilayer Metasurfaces
Source: Nanophotonics. 2026 Jan 16;15(2):e70012. doi: 10.1002/nap2.70012 (PMC12964997; doi:10.1002/nap2.70012)
Supplement: Supplementary file 1 — Supporting Information S1 [file NAP2-15-e70012-s001.docx]

Supporting Information

Phase Modulation for Quadruplex Channels of Arbitrary Orthogonal Polarization States via Bilayer Metasurfaces

Wei Wang, Jun Wang, Qiaohua Wu, Jie Lin, Peng Jin, Shutian Liu, Zhongyi Guo, and Keya Zhou**^∗^**

W. Wang, J. Wang, Q. Wu, J. Lin, S. Liu, K. Zhou

School of Physics, Harbin Institute of Technology, Harbin 150001, China

E-mail: [zhoukeya@hit.edu.cn](mailto:zhoukeya@hit.edu.cn)

J. Lin, P. Jin

Key Laboratory of Micro-systems and Micro-structures Manufacturing, Harbin Institute of Technology, Ministry of Education, Harbin 150080, China

P. Jin

School of Instrumentation Science and Engineering, Harbin Institute of Technology, Harbin 150001, China

Z. Guo

School of Computer and Information, Hefei University of Technology, Hefei 230009, China

**Note S1: Phase modulation for quadruplex channels via bilayer metasurfaces**

Firstly, consider the modulation of circularly polarized light by single-layer meta-atoms. Assuming that the meta-atoms are lossless, their Jones matrix can be expressed as:^[S1]^

where , , $I=\left[\begin{matrix} 1 & 0 \\ 0 & 1 \end{matrix}\right]$, $\hat{\sigma}_{\text{R}}=\frac{1}{2}\left[\begin{matrix} 1 & -i \\ -i & -1 \end{matrix}\right]$ and $\hat{\sigma}_{\text{L}}=\frac{1}{2}\left[\begin{matrix} 1 & i \\ i & -1 \end{matrix}\right]$. $I$, $\hat{\sigma}_{\text{R}}$, and $\hat{\sigma}_{\text{L}}$ have the following properties:

$$\left\{\begin{matrix} I|L\rangle=|L\rangle\\ I|R\rangle=|R\rangle\\ \hat{\sigma}_{\text{R}}|L\rangle=|R\rangle\\ \hat{\sigma}_{\text{R}}|R\rangle=0 \\ \hat{\sigma}_{\text{L}}|L\rangle=0 \\ \hat{\sigma}_{\text{L}}|R\rangle=|L\rangle\end{matrix}\right.$$

Notice that the role of $I$ is to keep the circular polarization state unchanged (convert to orthogonal complex conjugate state), and the role of $\hat{\sigma}_{\text{R}}$ and $\hat{\sigma}_{\text{L}}$ is to convert the input circular polarization state to its orthogonal state of polarization (convert to complex conjugate state).

Under the incomplete polarization conversion condition, the modulation of the orthogonal states of polarization ($|\lambda^{+}\rangle$ with $|\lambda^{-}\rangle$) by the metasurfaces can be expressed as:

$$\left\{\begin{matrix} M|\lambda^{+}\rangle=f_{1}|\lambda^{-}\rangle^{*}+f_{2}|\lambda^{+}\rangle^{*} \\ M|\lambda^{-}\rangle=f_{3}|\lambda^{-}\rangle^{*}+f_{4}|\lambda^{+}\rangle^{*} \end{matrix}\right.$$

here $f$ is the complex amplitude distribution. From Equation (S1) and (S3), there is $f_{1}=f_{4}$ for metasurfaces consisting of single-layer meta-atoms.

Referring to the properties of $I$, $\hat{\sigma}_{\text{R}}$ and $\hat{\sigma}_{\text{L}}$, the operators $\hat{\sigma}_{1}$ and $\hat{\sigma}_{2}$ are defined to convert the input polarization states into orthogonal complex conjugate states and complex conjugate states:

$$\left\{\begin{matrix} \hat{\sigma}_{1}|\lambda^{+}\rangle=f_{1}|\lambda^{-}\rangle^{*} \\ \hat{\sigma}_{1}|\lambda^{-}\rangle=f_{4}|\lambda^{+}\rangle^{*} \\ \hat{\sigma}_{2}|\lambda^{+}\rangle=f_{2}|\lambda^{+}\rangle^{*} \\ \hat{\sigma}_{2}|\lambda^{-}\rangle=f_{3}|\lambda^{-}\rangle^{*} \end{matrix}\right.$$

Thus, the matrix $M$ can be written as:

$$M=\hat{\sigma}_{1}+\hat{\sigma}_{2}$$

The incident orthogonal states of polarization $|\lambda^{+}\rangle$ and $|\lambda^{-}\rangle$ are defined as:

here, *A* and *B* denote the amplitude of the *x*- and *y*-polarized components of the electric field ($A^{2}+B^{2}=1$), respectively, and is the corresponding phase delay. Substituting Equation (S6) into Equation (S4) leads to:

The C1 and C4 channels have the same response ($f_{1}=f_{4}$). We consider using bilayer metasurfaces (Figure 1) in which the meta-atoms of one of the layers are equivalent to half-wave plates (HWPs) to realize the four-channel phase modulation. First, the bottom nanopillars are treated as HWPs ($a_{1}=0$), and the operator for the meta-atoms consisting of bilayer nanopillars is given by Equation (S1) as:

$$M=M_{2}\cdot M_{1}=\left(\frac{a_{2}}{2}I+\frac{b_{2}}{2}\hat{\sigma}_{\text{R}}e^{2i\theta_{2}}+\frac{b_{2}}{2}\hat{\sigma}_{\text{L}}e^{-2i\theta_{2}}\right)\cdot\left(\frac{b_{1}}{2}\hat{\sigma}_{\text{R}}e^{2i\theta_{1}}+\frac{b_{1}}{2}\hat{\sigma}_{\text{L}}e^{-2i\theta_{1}}\right)$$

here, the subscripts 1 and 2 indicate the bottom and top nanopillars. Combining Equation (S5), (S7) and (S8), the following Equation can be obtained:

It is clear that *f*_1_, *f*_2_, *f*_3_, and *f*_4_ are not identical to each other. According to the main text, we next consider several specific orthogonal states of polarization.

1. Orthogonal circular polarization states ($A=B=\frac{\sqrt{2}}{2}$, , $|\lambda^{+}\rangle=\frac{\sqrt{2}}{2}\left[\begin{matrix} 1 \\ i \end{matrix}\right]$, $|\lambda^{-}\rangle=\frac{\sqrt{2}}{2}\left[\begin{matrix} 1 \\ -i \end{matrix}\right]$)

Equation (S9) can be simplified as:

where and .

1. Orthogonal linear polarization states ($A=1$, $B=0$, , $|\lambda^{+}\rangle=\left[\begin{matrix} 1 \\ 0 \end{matrix}\right]$, $|\lambda^{-}\rangle=\left[\begin{matrix} 0 \\ 1 \end{matrix}\right]$)

Equation (S9) can be simplified as:

1. Orthogonal elliptical polarization states ($A=\frac{\sqrt{3}}{2}$, $B=\frac{1}{2}$, , $|\lambda^{+}\rangle=\frac{1}{2}\left[\begin{matrix} \sqrt{3} \\ i \end{matrix}\right]$, $|\lambda^{-}\rangle=\frac{1}{2}\left[\begin{matrix} 1 \\ -\sqrt{3}i \end{matrix}\right]$)

Equation (S9) can be expressed as:

1. Orthogonal first-order cylindrical vertical polarization states ($A=\cos\beta$, $B=\sin\beta$, , $|\lambda^{+}\rangle=\left[\begin{matrix} \cos\beta\\ \sin\beta\end{matrix}\right]$, $|\lambda^{-}\rangle=\left[\begin{matrix} \sin\beta\\ -\cos\beta\end{matrix}\right]$), here $\beta$ is azimuth angle.

Equation (S9) can be expressed as:

If the top nanopillars are treated as half-wave plates ($a_{2}=0$), the operator of the meta-atoms consisting of bilayer nanopillars is given by Equation (S1) as:

$$M=M_{2}\cdot M_{1}=\left(\frac{b_{2}}{2}\hat{\sigma}_{\text{R}}e^{2i\theta_{2}}+\frac{b_{2}}{2}\hat{\sigma}_{\text{L}}e^{-2i\theta_{2}}\right)\cdot\left(\frac{a_{1}}{2}I+\frac{b_{1}}{2}\hat{\sigma}_{\text{R}}e^{2i\theta_{1}}+\frac{b_{1}}{2}\hat{\sigma}_{\text{L}}e^{-2i\theta_{1}}\right)$$

By combining Equations (S5), (S7), and (S14), we can obtain:

Similar to Equation (S9), this design also enables four-channel phase modulation. For example, when orthogonally circularly polarized beams are incident, Equation (S15) can be simplified as:

**Note S2: Simulation of meta-atoms**

The designed meta-atom is shown in Figure 1c with a period of 360. The design wavelength is 532 nm. We neglect the coupling between the bottom and top nanopillars and directly scan the structural parameters of the single-layer nanopillars using the rigorous coupled wave analysis (RCWA) method with a step of 5 nm.^[S2]^ The refractive indices of SiO_2_ and TiO_2_ are 1.461 and 2.314, respectively.^[S3,S4]^ The transmission and phase delay of the incident *x*-polarized light are shown in Figure S1a, and the transmissions and phase delays of the 20 selected meta-atoms acting as HWPs are shown in Figure S1b.


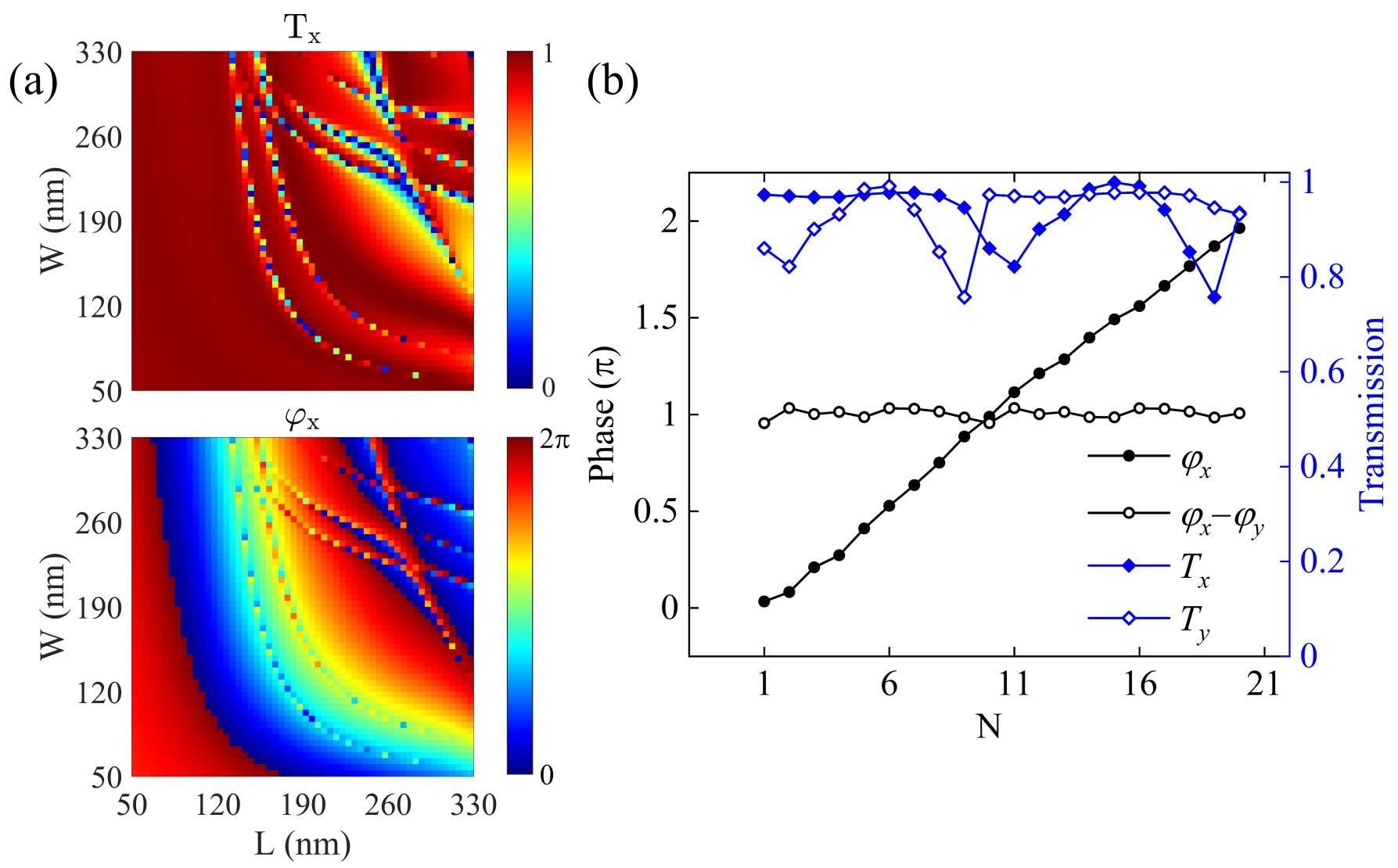


**Figure S1.** (a) Transmission and phase delay maps of meta-atoms with different dimensions obtained by the RCWA method. (b) Transmissions and phase delays of the 20 selected meta-atoms acting as HWPs.

Here, we introduce the method for determining the meta-atom parameters ($\theta_{1}$, $\theta_{2}$ , , and ) of the various metasurfaces designed in this work. First, the theoretical parameters $\theta_{1}$, $\theta_{2}$ , and of meta-atoms are calculated based on the complex amplitudes (*f*_1_, *f*_2_, *f*_3_, and *f*_4_) of the four channels.

1. Metasurfaces for controlling circularly polarized light.

According to Equation (S10), the phases and rotation angles of the meta-atoms can be expressed as

Here, $\text{abs}\left(.\right)$ and $\text{ang}\left(.\right)$ denote the operations for extracting amplitude and phase, respectively. We treat and as a whole ().

1. Metasurfaces for controlling linearly polarized light.

According to Equation (S11), the phases and rotation angles of the meta-atoms can be expressed as

Here, $\text{real}\left(.\right)$ and $\text{imag}\left(.\right)$ denote the operations for extracting the real part and the imaginary part, respectively.

1. Metasurfaces for controlling elliptically polarized light.

According to Equation (S12), the phases and rotation angles of the meta-atoms can be expressed as

1. Metasurfaces for controlling first-order cylindrically vectorially polarized light.

According to Equation (S13), the phases and rotation angles of the meta-atoms can be expressed as

Subsequently, appropriate structures are selected from the meta-atom library (Figure S1) based on the calculated parameters and . We first select top structures from the data shown in Figure S1a using the criterions , $\text{T}_{xx}>0.9$, and $\text{T}_{yy}>0.9$, where , , $\text{T}_{xx}$, and $\text{T}_{yy}$ represent the actual phase delays and transmissions of the structures. When the data meets the criterions, we record the height and width of the structure corresponding to that data. If no data satisfy the criterions, the criterions is progressively relaxed until data that meets the conditions exists. Next, the bottom HWP structures are selected such that the is minimized, here, represents the phase delay of the HWP structure. Similarly, when the data meets the criteria, we record the length and width of the corresponding HWP structure. The screening process for bilayer nanopillars at all positions is identical. Finally, we arrange the nanopillars based on their coordinates and the selected structural parameters to form the metasurfaces.

**Note S3: Design and simulation of metasurfaces**

This work demonstrates 11 metasurfaces (noted as M1, M2, M3, M4, M5, M6, M7, M8, M9, M10 and M11, respectively) with different functions. The design methods and related parameters are as follows:

**Note S3.1: Metasurfaces for orbital angular momentum (OAM)** **manipulation (M1 and M2)**

M1 and M2 with apertures of 36 μm are used to realize OAM manipulation of orthogonal circular and linear polarization quadruplex channels, respectively. The corresponding four-channel complex amplitude distributions are described by Equation (S10) and (S11), respectively. The amplitudes of the quadruplex channels (C1, C2, C3, and C4) corresponding to *f*_1_, *f*_2_, *f*_3_ and *f*_4_ are $\sqrt{2}/2$ and the phase distributions are:

where $\beta=\arctan\frac{y}{x}$ is the azimuth angle, $\lambda$ denote the design wavelength, and the focal length *f* is 40 μm. The topological charges $l_{i}$ of the quadruplex channels are -2, 2, -1, and 3, respectively.

The OAM purity and intensity distributions of the vortex beams in the four channels generated by M1 and M2 are shown in Figures S2 and S3, respectively. The focusing efficiencies of the vortex beams in the four channels of M1 are 32.26%, 33.72%, 32.39%, and 32.8%, respectively. And the focusing efficiencies of the vortex beams in the four channels of M2 are 32.53%, 30.82%, 30.21%, and 32.95%, respectively. Focusing efficiency is the ratio of energy within the focused area to the energy of the incident light, the focused area is defined as a circular area centered on the focal spot. The diameter of the vortex light's focused area is three times that of the annular spot (indicated by the blue arrows in Figure S2b). The diameter of the focused area of the Gaussian light is three times the full width at half maximum (FWHM).


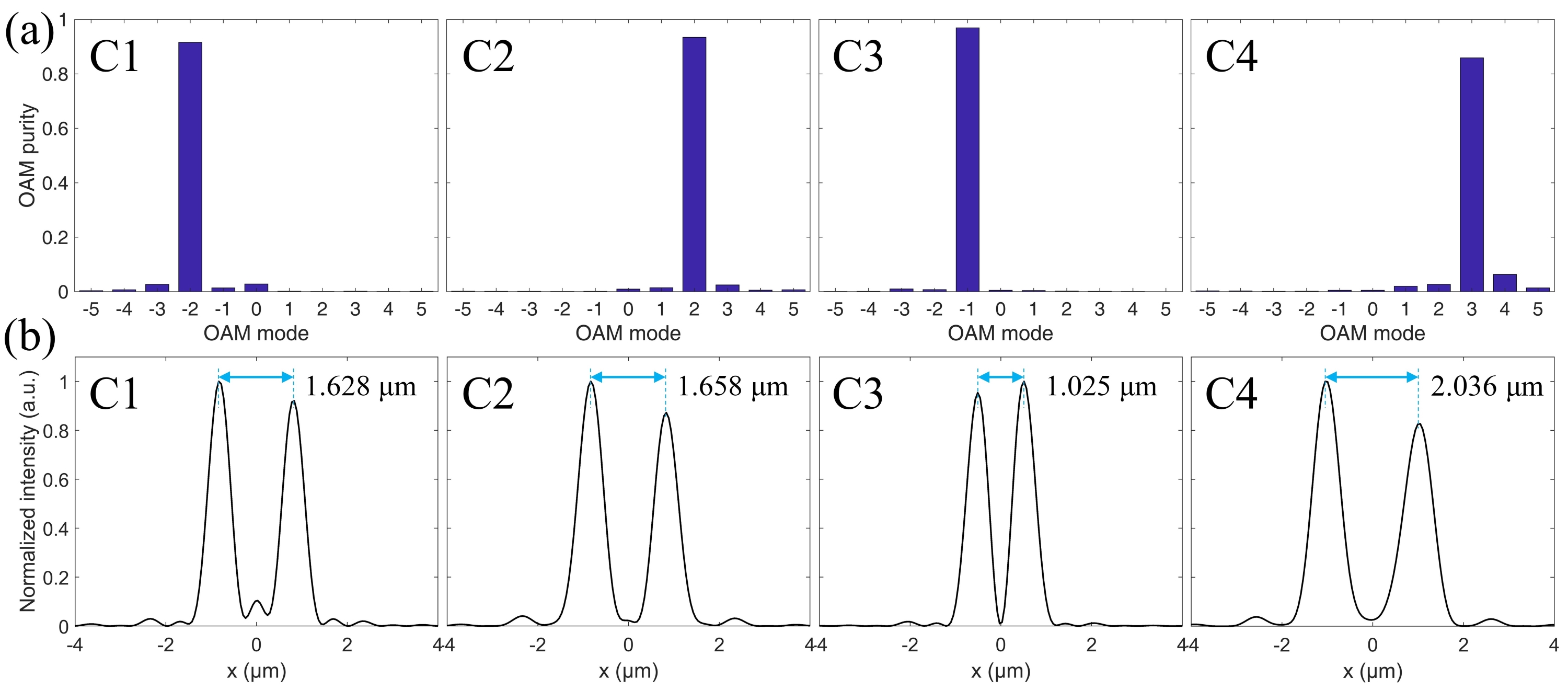


**Figure S2.** OAM purity (a) and intensity distributions (b) of focused vortex beams generated by M1. The transverse electric field distributions at 40 μm was utilized to calculate the OAM purity and plot the intensity distributions.


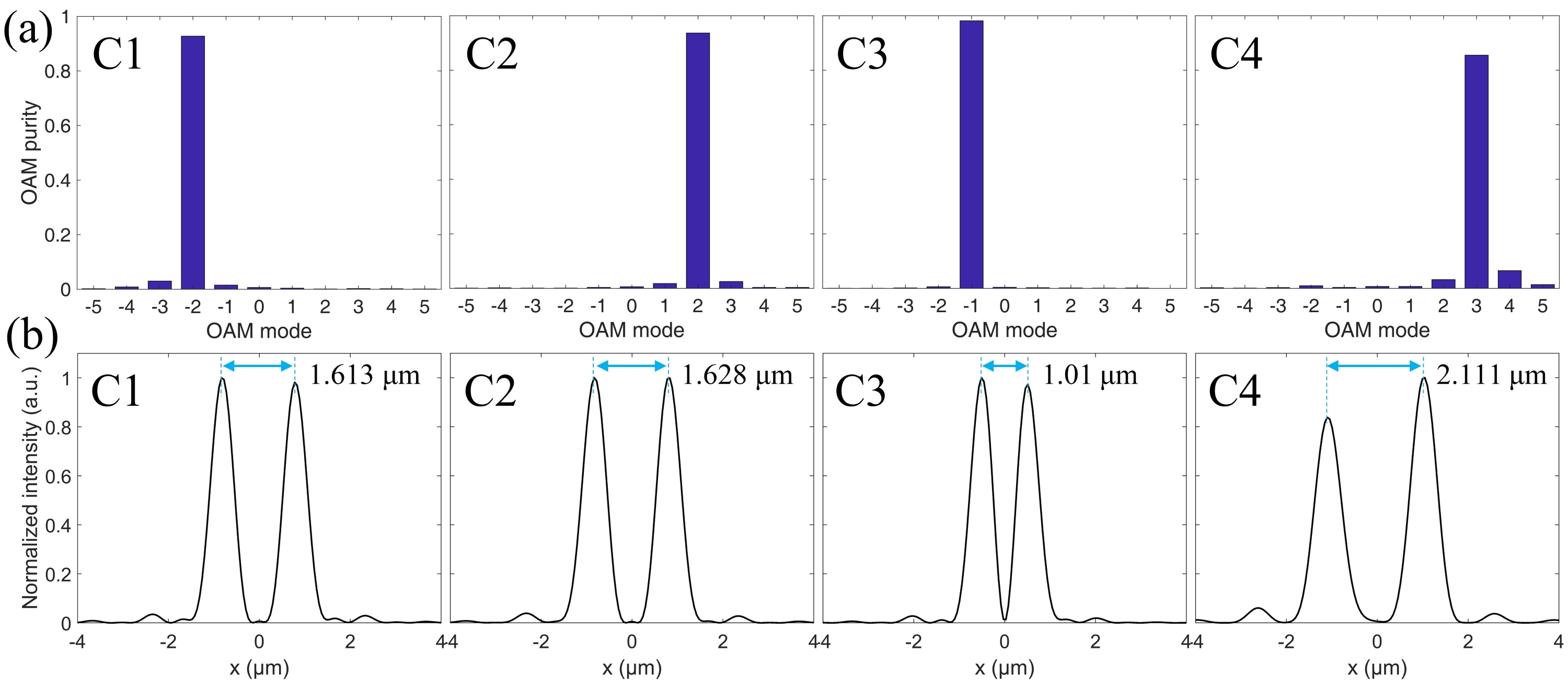


**Figure S3.** OAM purity (a) and intensity distributions (b) of focused vortex beams generated by M2. The transverse electric field distributions at 40 μm was utilized to calculate the OAM purity and plot the intensity distributions.

**Note S3.2: Metasurface for generating Bessel beams (M3)**

The M3 with 36 μm aperture is used to modulate the longitudinal wavevectors and topological charges of the four-channel Bessel beams. Equation (S10) guides the design of the M3. The phase distributions of the quadruplex channels are:

The numerical aperture (NA) is used to control the non-diffracting lengths (longitudinal wavevector) of the Bessel beam. The numerical apertures of the quadruplex channels are 0.3, 0.25, 0.22, and 0.17, and the topological charges are 0, 2, -1, and 1, respectively.

The cylindrical vector vortex beam can be decomposed into superimposed orthogonally circularly polarized vortex beams:^[S5]^

$$e^{il\beta}\left[\begin{matrix} \cos\left(m\beta+\beta_{0}\right) \\ \cos\left(m\beta+\beta_{0}\right) \end{matrix}\right]=\frac{1}{2}e^{i\left(l_{\text{R}}+\beta_{0}\right)}\left[\begin{matrix} 1 \\ -i \end{matrix}\right]+\frac{1}{2}e^{i\left(l_{\text{L}}-\beta_{0}\right)}\left[\begin{matrix} 1 \\ i \end{matrix}\right]$$

where $m=\left(l_{\text{R}}-l_{\text{L}}\right)/2$ and $l=\left(l_{\text{R}}+l_{\text{L}}\right)/2$ denote the polarization order and topological charge of the vector vortex beam, respectively. From Equation (S23), the polarization distribution of the vector vortex beam produced by the superposition varies along the propagation direction if the phase difference $2\beta_{0}$ between the left- and right-handed circularly polarized light varies along the propagation direction. The longitudinally varying phase difference can be achieved by superimposing orthogonally circularly polarized light with different longitudinal wavevectors. Thus, when left- or right-handed circularly polarized vortex light irradiates M3, the resulting orthogonally circularly polarized Bessel beams are superimposed as vector Bessel beams with longitudinally varying polarization states. In addition, according to Equation (S23), the polarization orders of the Bessel beams generated by M3 illuminated by left- or right-handed circularly polarized light are both 1, and the topological charges are 1 and 0, respectively. The corresponding intensity distributions at different longitudinal locations are shown in Figure 3c.

The OAM purity and intensity distributions of the Bessel beams in the four channels generated by M3 are shown in Figures S4. The generation efficiencies of the four-channel Bessel beams are 4%, 14.63%, 8.31%, and 15.38%, respectively. Here, the generation efficiency is defined as the ratio of the energy in the main lobe of the Bessel beam to the incident light energy. The longitudinal intensity distributions plotted based on the main lobes of the Bessel beams are shown in Figure S5. With a threshold of 0.1, the non-diffracting lengths of the four-channel Bessel beams are close to 56.51 μm, 63.85 μm, 73.88 μm, and 103.58 μm, which are similar to the values (57.24 μm, 69.71 μm, 79.81 μm, and 104.34 μm) calculated based on $D/\left(2\tan\left(\text{asin}\left(NA\right)\right)\right)$,^[45]^ where $D=36\text{ μm}$ is the diameter of the metasurface.


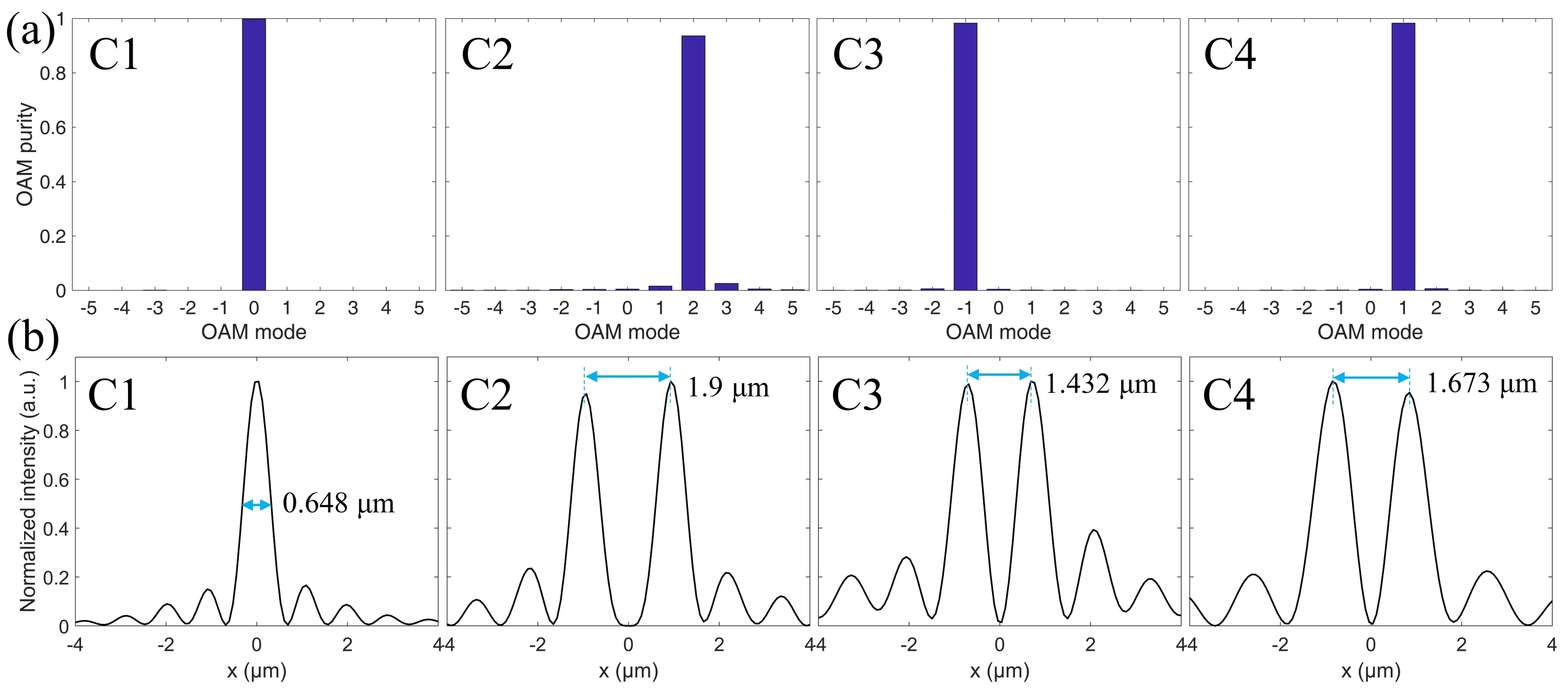


**Figure S4.** OAM purity (a) and intensity distributions (b) of Bessel beams generated by M3. The transverse electric field distributions at 50 μm and 70 μm (white dotted lines in Figure 3a) were used to calculate the OAM purity and plot the intensity distribution for the four channels.


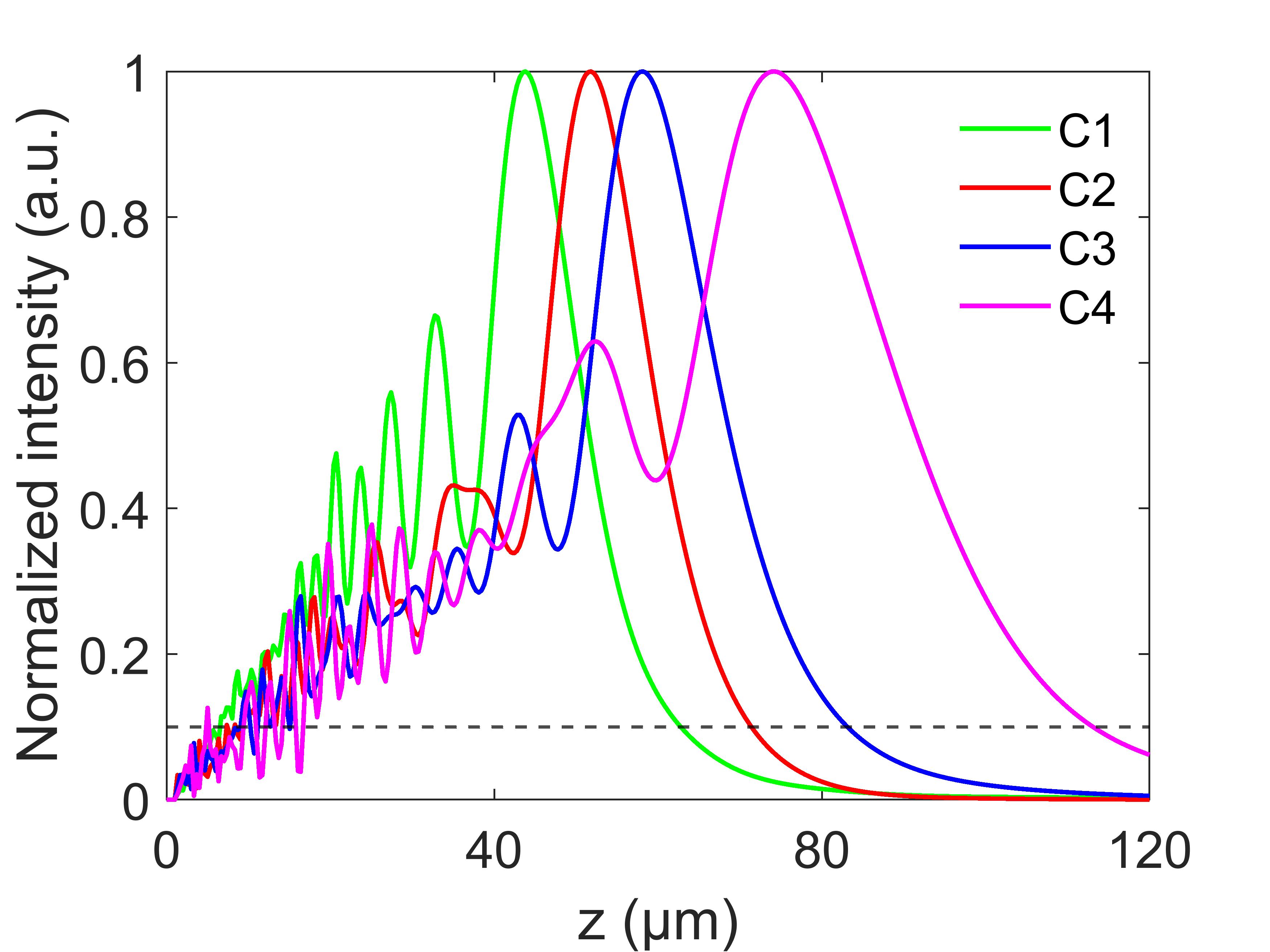


**Figure S5.** Longitudinal intensity distributions of the Bessel beams.

**Note S3.3: Gradient metasurface (M4)**

The phase gradients along the *x* direction of the designed gradient metasurface M4 are expressed as:

where *P* is the period of the meta-atoms and $N=8$ is the number of meta-atoms along the *x* direction within the supercell. The diffraction orders $m_{i}$ of the quadruplex channels are 2, -1, 3, and 0, respectively. The meta-atoms are selected according to Equation (S11). When simulating the supercell by the finite-difference time domain (FDTD) method, periodic boundary condition is set in the *x* and *y* directions, and perfectly matched layer (PML) boundary condition is set in the *z* direction. The simulated diffraction efficiencies of 2, -1, 3, and 0 diffraction orders are 41.67%, 41.38%, 31.67%, and 29.74%, respectively.

**Note S3.4: Metasurface for four-channel holography (M5)**

M5 is used to generate four holographic images (four strings L, R, HIT, and meta) within orthogonally elliptically polarized ($\left[\begin{matrix} \sqrt{3}/2 & i/2 \end{matrix}\right]^{\text{T}}$ and $\left[\begin{matrix} -i/2 & -\sqrt{3}/2 \end{matrix}\right]^{\text{T}}$) quadruplex channels. The dimension of the M5 is 72×72 μm. The original pictures of the strings L, R, HIT, and meta are shown in Figure S6a. The corresponding phase distributions of the original pictures are obtained by the Gerchberg-Saxton (G-S) algorithm, as shown in Figure S6b.

Equation (S12) guides the design of M5. As emphasized in the main text, the completely decoupled control of four-channel phases is difficult to achieve in most cases. Because Equation (S9) indicates that the phases of the quadruplex channels satisfy:

Here, our solution is to add or subtract small phase deviations to the target phases of the four channels, such that the modified phases satisfy Equation (S25). The specific steps are as follows:

1. Set weights $w$ for the four-channel phases: $w_{Ci}=1$ ($i=1,2,3,4$). Normalized weights:

$$w_{Ci}=\frac{w_{Ci}}{\sum w_{Ci}}$$

1. Calculate the deviation $d$ between the target phases and the constraint condition:

where ($i=1,2,3,4$) is the target phase.

1. Calculate the phases satisfying the constraint condition:

1. Calculate , , , $\theta_{1}$, and $\theta_{2}$ based on the modified phases and Equation (S12).

The adjusted phase distributions calculated through the above steps are presented in Figure S6c. These calculation steps are also used to calculate the phases of the metasurfaces afterwards. As expected, this approach leads to reduced metasurface efficiency under incomplete decoupling conditions. Compared to M1 and M2 (complete decoupling), the focusing efficiency of metasurfaces M6, M7, M8, M9, M10 and M11 decreases by approximately 30%. This problem can be alleviated by optimization algorithms.^[30, 42]^

The generation efficiencies (the energy ratio between the holographic image and the incident light) of the holograms for the four channels are 15.83%, 15.94%, 14.45%, and 15.57%, respectively.


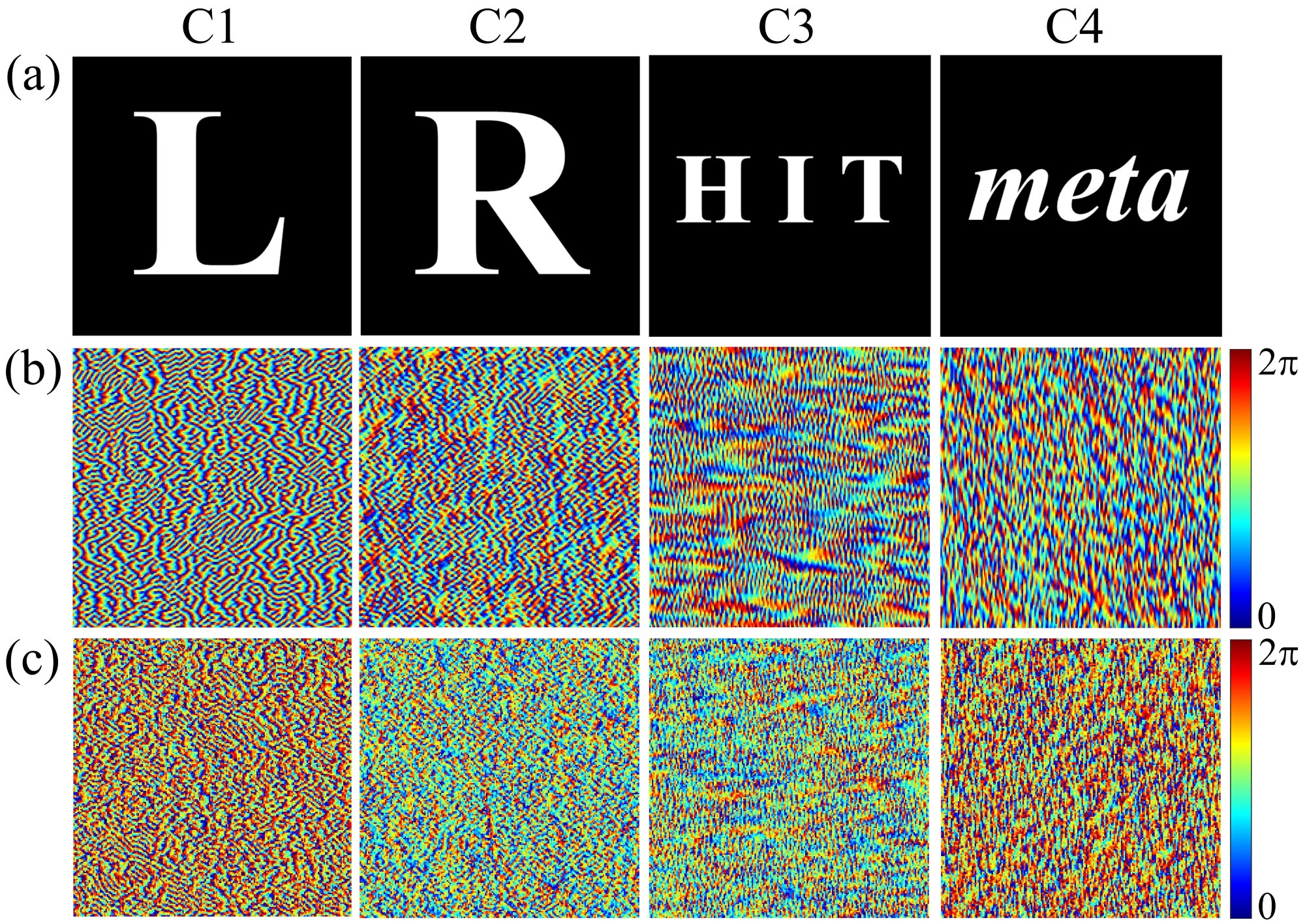


**Figure S6.** (a) Original hologram images of elliptical polarization quadruplex channels. (b) Phase distributions obtained by the G-S algorithm. (c) The phase distributions that satisfy the constraint condition (Equation (S25)).

**Note S3.5: Metasurface for modulating vectorially polarized light (M6)**

The dimension of M6 is 72×72 μm. M6 produces focused radially and azimuthally polarized light when illuminated by radially polarized light. The M6 is designed based on Equation (S13), the phase distributions of the C1 and C2 channels are:

where $f=20\text{ μm}$, $x_{1}=2\text{ μm}$, $x_{2}=-2\text{ μm}$. M6 generates dot matrix holographic images (numbers 0 and 1) when illuminated by azimuthally polarized light, and 0 and 1 are encoded into azimuthal polarization and radial polarization, respectively. The design process for the phase distributions of the C3 and C4 channels is shown in Figure S7, where $d=2.88\text{ μm}$.

The complete transverse distributions of the *x*- and *y*-polarized component intensities of the electric field of the C3 and C4 channels are shown in Figure S8. The noise points and uneven intensity distribution in holograms are caused by deviations of transmission and phase. The generation efficiencies of holograms for channels C3 and C4 are 13.73% and 13.29%, respectively. The intensity distributions of focused radially and azimuthally polarized beams are shown in Figure S9, with focusing efficiencies of 20.86% and 22.45%, respectively.


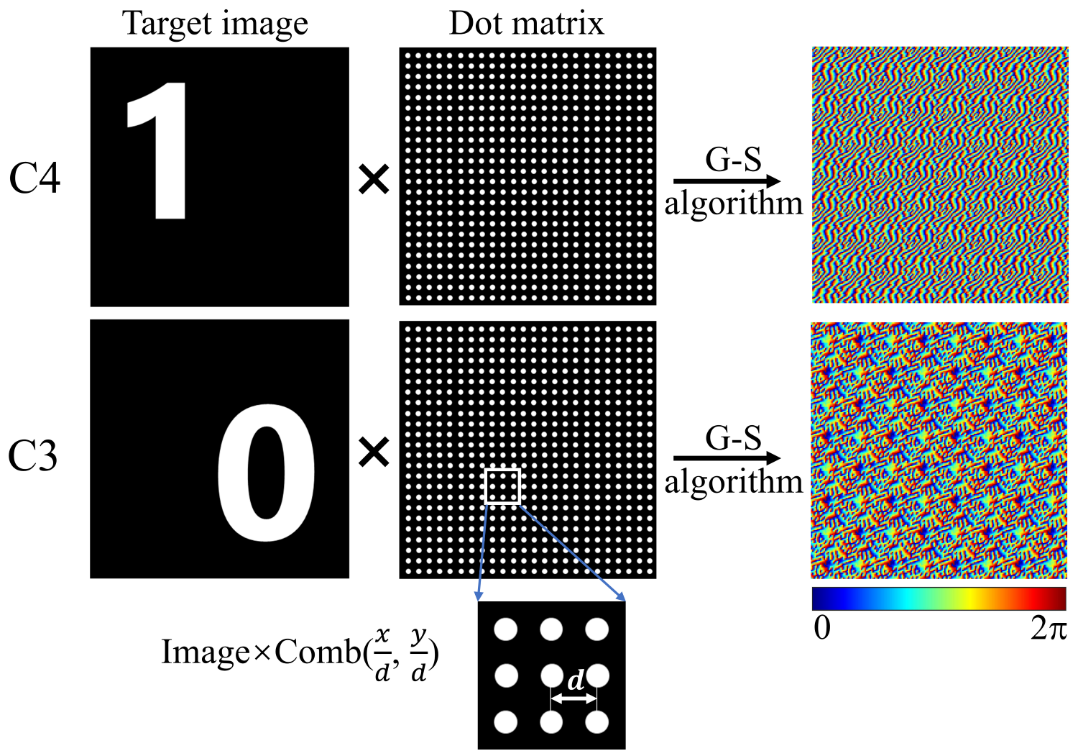


**Figure S7.** Design process for phase distributions of vector dot matrix holographic images.


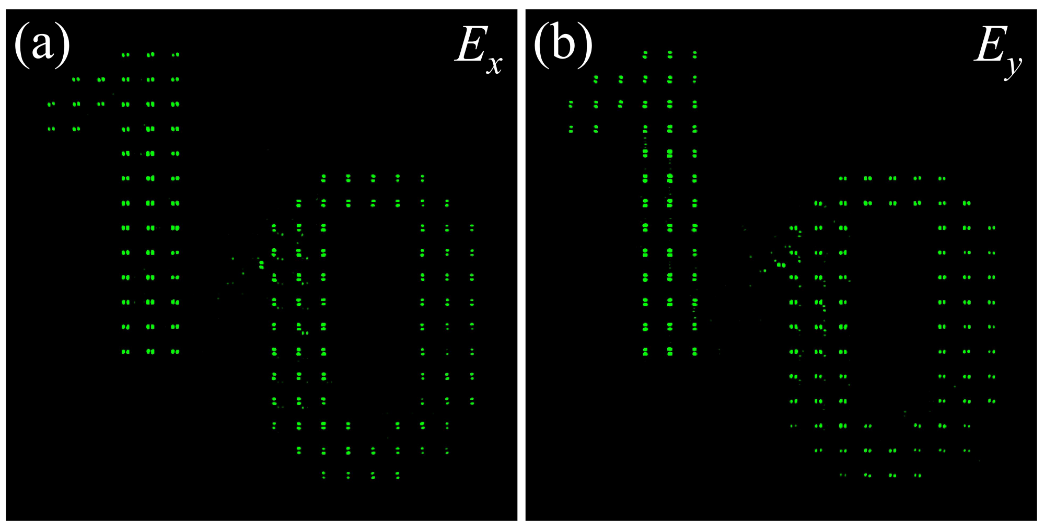


**Figure S8.** Intensity distributions of the *x* and *y* polarized components of the electric field in the C3 and C4 channels.


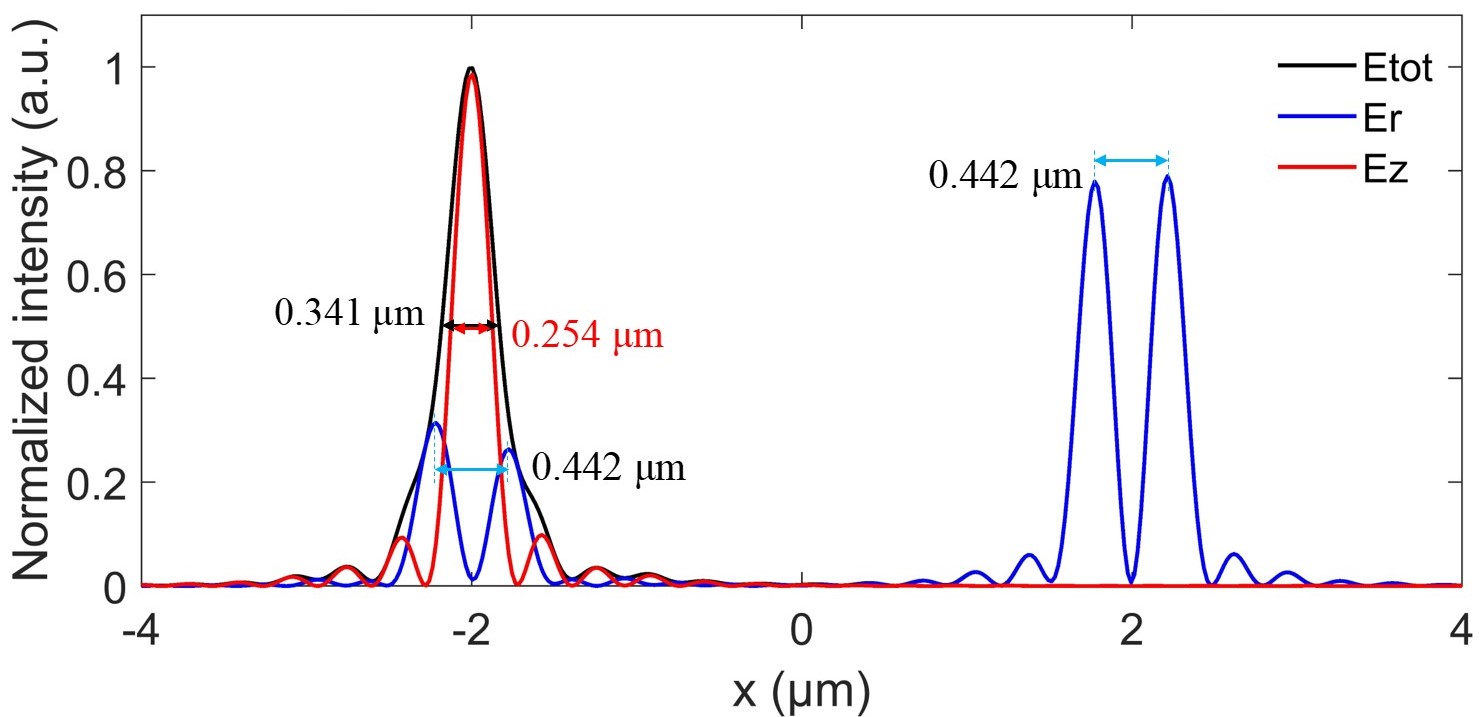


**Figure S9.** The intensity distributions of the separated tightly focused radially and azimuthally polarized beams.

**Note S3.6: Metasurfaces for four-channel energy modulation (M7, M8, M9, M10, M11)**

To demonstrate that the proposed strategy can control the energy ratios of different channels, we design five metasurfaces (M7, M8, M9, M10, and M11) with a 36 μm aperture according to Equation (S12). The energy ratios of the C1 and C2 channels (or C4 and C3 channels) of the five metasurfaces are designed as 1:0, 2:1, 1:1, 1:2, and 0:1, respectively. The phase distributions of the quadruplex channels are expressed as:

where $f=40\text{ μm}$, $\left(x_{i},y_{i}\right)$ denotes the focal coordinates. The focal coordinates of the C1, C2, C3 and C4 polarization channels are $\left(\text{3 μm},0\right)$, $\left(-\text{3 μm},0\right)$, $\left(0,4\text{ μm}\right)$, and $\left(0,-4\text{ μm}\right)$, respectively. The focusing efficiencies (η) and the FWHMs of the focused light spots for the four channels are shown in Table S1.

**Table S1.** Four-channel focusing efficiencies (η) and FWHMs.

|  | C1 | | C2 | | C3 | | C4 | |
| --- | --- | --- | --- | --- | --- | --- | --- | --- |
|  | η  (%) | FWHM  (μm) | η  (%) | FWHM  (μm) | η  (%) | FWHM  (μm) | η  (%) | FWHM  (μm) |
| M7 | 45.57 | 0.6602 | / | | / | | 46.17 | 0.6582 |
| M8 | 29.54 | 0.6575 | 13.65 | 0.6896 | 14.15 | 0.6815 | 29.4 | 0.6536 |
| M9 | 21.99 | 0.6622 | 21.46 | 0.6806 | 19.79 | 0.6742 | 21.89 | 0.6569 |
| M10 | 14.46 | 0.6633 | 29.16 | 0.6751 | 30.02 | 0.6712 | 13.84 | 0.658 |
| M11 | / | | 42.4 | 0.6711 | 41.11 | 0.6687 | / | |

Next, we will discuss the accuracy, linearity, and loss of energy regulation. First, we define $R=\text{E}_{\text{C1}}/\text{E}_{\text{C2}}$ and $R^{*}=\text{E}_{\text{C4}}/\text{E}_{\text{C3}}$, where $\text{E}_{\text{C1}}$, $\text{E}_{\text{C2}}$, $\text{E}_{\text{C3}}$, and $\text{E}_{\text{C4}}$ represent the total energies of channels C1, C2, C3, and C4, respectively. Equations (S9) and (3) show that *R* = *R*^*^. Based on theory and simulation results, we obtain Table S2.

**Table S2.** Accuracy of energy regulation for the designed metasurfaces.

|  |  | | M7 | M8 | M9 | M10 | M11 |
| --- | --- | --- | --- | --- | --- | --- | --- |
| *R* | Theory | | 1:0 | 2:1 | 1:1 | 1:2 | 0:1 |
|  | Simulation | | 64.7895 | 2.1636 | 1.05 | 0.5191 | 0.0272 |
| *R*^*^ | Theory | | 1:0 | 2:1 | 1:1 | 1:2 | 0:1 |
|  | Simulation | | 67.9655 | 2.1221 | 1.0263 | 0.4888 | 0.0275 |
| Relative error of *R* | | | / | 8.18% | 5% | 3.81% | / |
| Relative error of *R*^*^ | | |  | 6.1% | 2.63% | -2.25% |  |
| *R*/*R*^*^ | | Theory | 1:1 | 1:1 | 1:1 | 1:1 | 1:1 |
|  |  | Simulation | 0.9533 | 1.0195 | 1.0231 | 1.062 | 0.9885 |
| Relative error of *R*/*R*^*^ | | | -4.67% | 1.95% | 2.31% | 6.2% | 1.15% |

Next, we demonstrate the linearity of energy regulation. The relationships between the energy proportions of channels C1, C2, C3, and C4 relative to the total output energies and the polarization conversion efficiency (PCE) are shown in Figure S10. Figure 5c is a composite of Figure S10. We fitted the data from the four channels, yielding the dotted curves shown in Figure S10. The corresponding fitting functions are:

$$\begin{matrix} \text{C}1:\text{ }f\left(x\right)=-0.9651x+0.9924 \\ \text{C2}:\text{ }f\left(x\right)=0.9651x+0.00762 \\ \text{C3}:\text{ }f\left(x\right)=0.9682x+0.01052 \\ \text{C4}:\text{ }f\left(x\right)=-0.9682x+0.9895 \end{matrix}$$

where $f\left(x\right)$ and *x* represent the PCE and energy proportion, respectively. The coefficients of determination (*R*^2^) calculated based on $R^{2}=1-\frac{SSE}{SST}$ are 0.9997, 0.9997, 0.9993, and 0.9993, respectively, where *SSE* and *SST* represent the sum of squared errors and the sum of squares total, respectively. We also calculated the Pearson correlation coefficients for the four channels, which are -0.9997, 0.9997, 0.9996, and -0.9996, respectively. The absolute values of these coefficients are close to 1, indicating an extremely strong linear correlation between the designed PCE and the energy proportion of each channel.


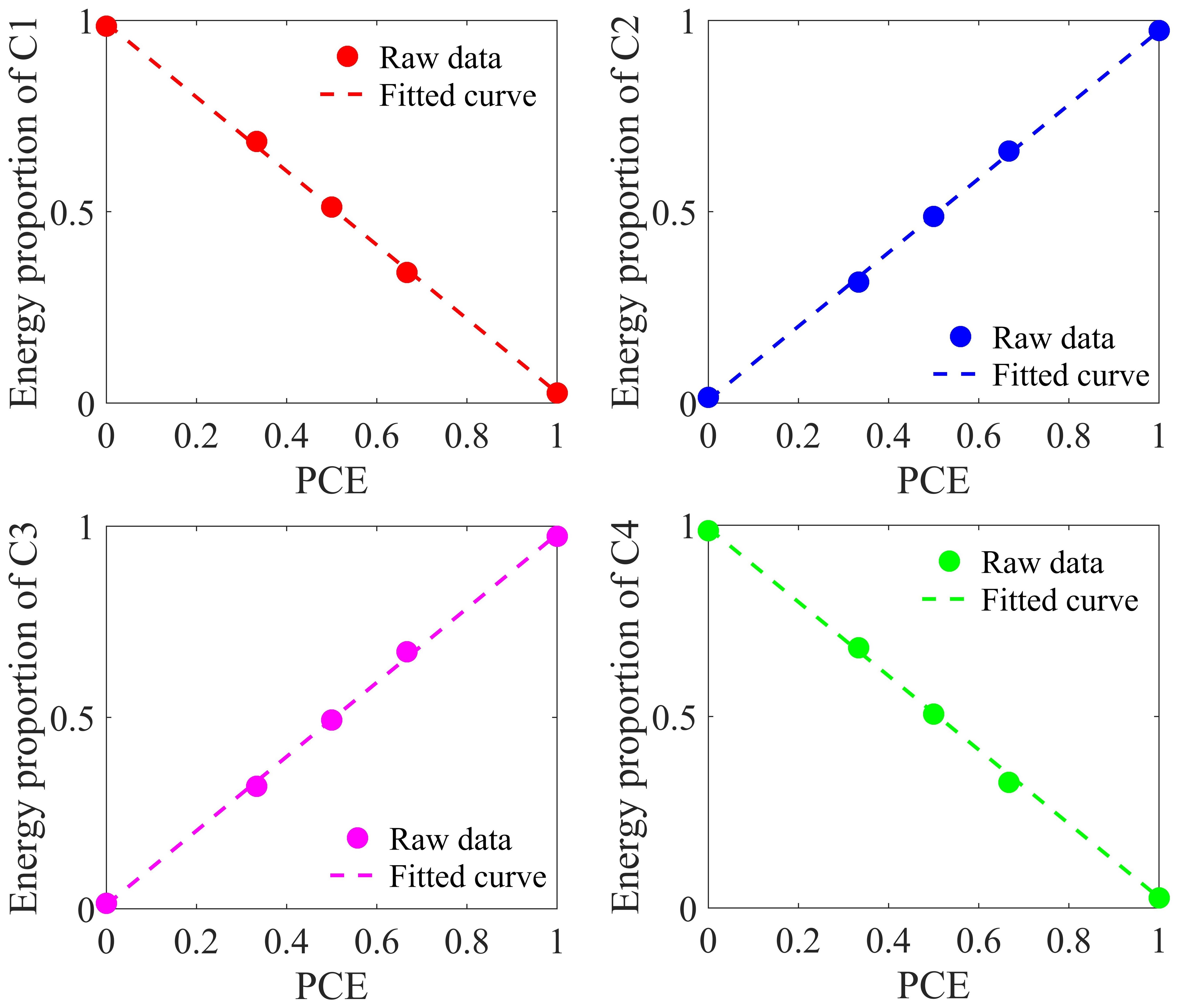


**Figure S10.** The proportions of the energies from different channels relative to the total output energies, and the fitted curves between PCEs and energy proportions.

Finally, we calculate the energy loss of the five metasurfaces. Figure S11 illustrates the proportions of energy in each channel relative to the total input energy, as well as the energy losses in each metasurface. The results indicate that the energy losses of the five metasurfaces do not exceed 35% of the input energy.


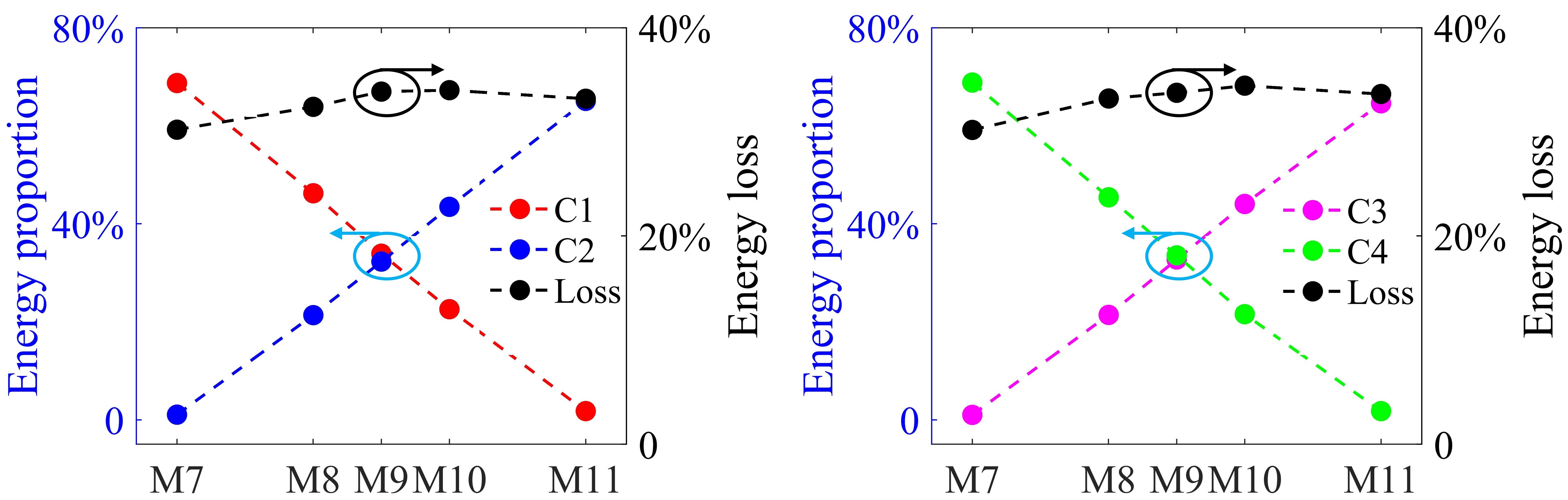


**Figure S11.** The proportions of energy in each channel relative to the total input energy, and the energy losses of each metasurface.

Theoretically, there is no limit to the value of the energy ratio. In addition to M7 ~ M11, we designed 10 metasurfaces here. The energy ratios *R* between the C1 and C2 channels for these metasurfaces are 1:5, 1:10, 1:20, 1:25, 1:30, 1:40, 1:50, 1:100, 1:200, and 1:400. Here, we only investigated the energy and intensity distributions of the C1 and C2 channels. Figure S12 shows the intensity profiles along the *x*-axis at the focal plane for different designed energy ratios *R*. The intensity profiles indicate that even with a design energy ratio *R* of 1:400 (red line), the beam from C1 channel can still be focused at the design focus $\left(3\text{ μm},\text{ 0 μm}\right)$.


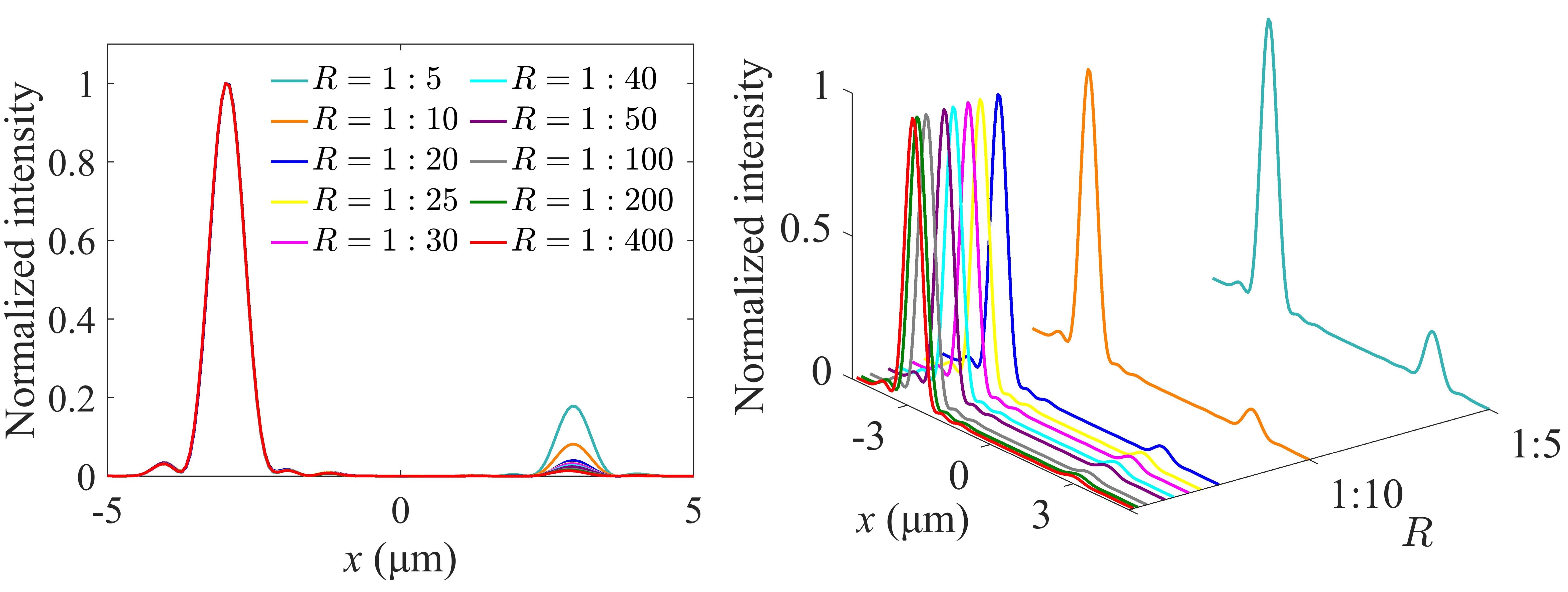


**Figure S12.** Intensity profiles along the *x*-axis for different design energy ratios *R*. The left and right figures present different perspectives and share the common legend.

However, simulation results indicate that when the designed energy ratio *R* exceeds 1:20, a significant deviation (>10%) emerges between the simulated and designed energy ratios, as shown in Figure S13. Figure S13a presents the simulated energy ratios (colored circles) and the designed energy ratio (gray line), with the vertical axis representing the energy ratio between C1 ($\text{E}_{\text{C1}}$) and C2 ($\text{E}_{\text{C2}}$) channels. Figure S13b illustrates the relative error between the simulated energy ratio and the designed energy ratio.


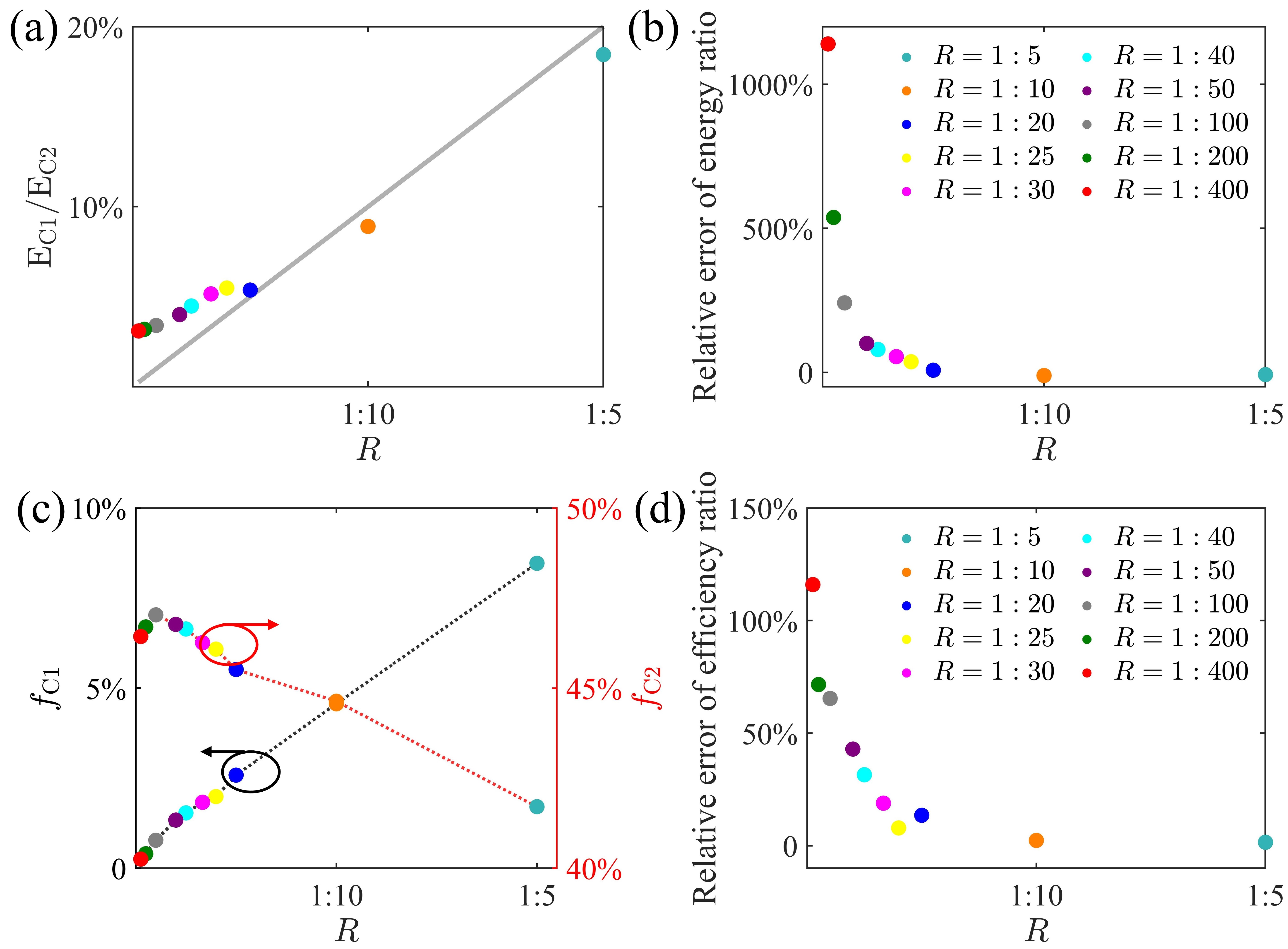


**Figure S13.** (a) Simulated energy ratios (colored circles) versus the designed energy ratio (gray line). (b) Relative error between the simulated and designed energy ratios. Both figures share the common legend.

We believe that when the designed energy ratio is low, both channels retain sufficient energy, resulting in reduced sensitivity of their energy ratio to noise. When the energy ratio is high, the energy in channel C1 is relatively low. In this case, the influence of noise on C1 cannot be neglected, which leads to a deviation between the simulated and designed energy ratios. In fact, the noise issue is also distinctly manifested in the M7 (or M11). Theoretically, the energy ratio between the C1 and C2 channels of the M7 (or M11) is 1:0 (or 0:1), meaning that the energy share of channel C2 (or C1) constitutes 0% of the total energy. However, simulation results indicate that the energy share of the C2 (or C1) channel for M7 (or M11) is 1.52% (or 2.65%) of the total energy.

We believe this unwanted noise is caused by transmission and phase deviations. This phase deviation has two sources. The first is the deviation between the target phase distribution and the approximate phase distribution. As stated in the Supporting Information Note S3.4, Equation (S9) does not have exact solutions in most cases. Therefore, we obtain the approximate phase distributions that satisfy Equation (S9) based on the target phase distributions of the four channels, which results in phase deviations. Second is the deviation between the approximate phase distributions and the phase responses of meta-atoms. As illustrated in Figure S1, we selected twenty half-wave plate nanopillars to cover the 2π phase range. Nevertheless, the deviation persists between the phases of these nanopillars and the approximate phases calculated from Equation (S12). Similarly, there is still a gap between the selected meta-atoms' transmission and the desired 100%, which can lead to unwanted non-uniform responses in the metasurfaces. Additionally, coupling between adjacent nanopillars can also cause the phase and amplitude responses of the meta-atoms to deviate from expectations.

**Note S4:** **Wideband operational characteristics of the metasurface**

To validate the broadband characteristics of the metasurfaces, the wavelength of incident light for M9 was varied from 490 nm to 630 nm in steps of 10 nm. Figure S14 shows the focal plane intensity distributions of the four channels at wavelengths of 490, 532, 570, and 610 nm. The energy ratios and focusing efficiencies of the four channels are shown in Figure S15. For ease of comparison, the energy ratios and focusing efficiencies at the design wavelength (532 nm) are used as the reference unit. It is evident that the variation in relative energy ratio does not exceed 10% within the simulated wavelength range. Furthermore, the relative focusing efficiencies of the four channels are higher than 80% in the range of 510 ~ 590 nm. Therefore, the designed metasurface demonstrates robust performance over an 80 nm wavelength range.


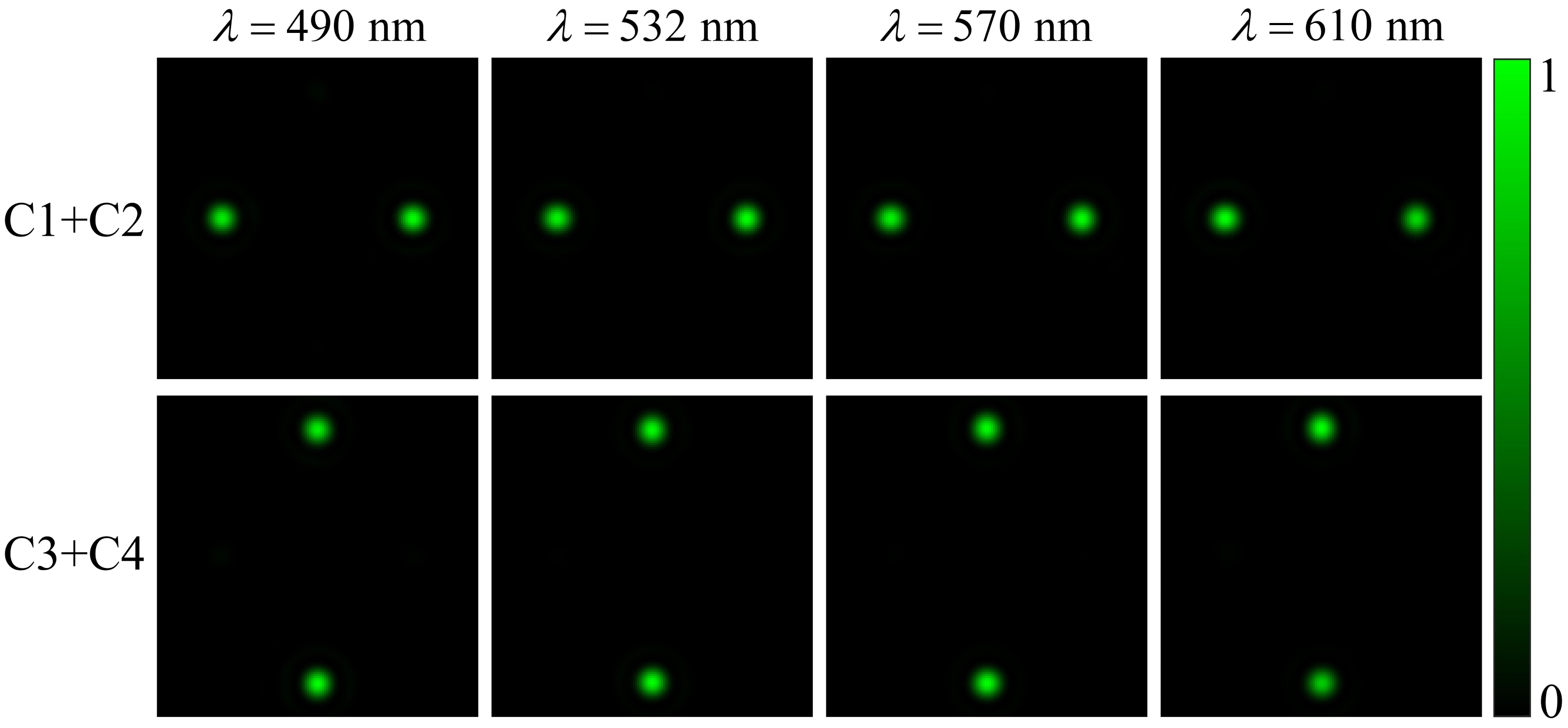


**Figure S14.** Focal plane intensity distributions of the four channels at wavelengths of 490, 532, 570, and 610 nm.


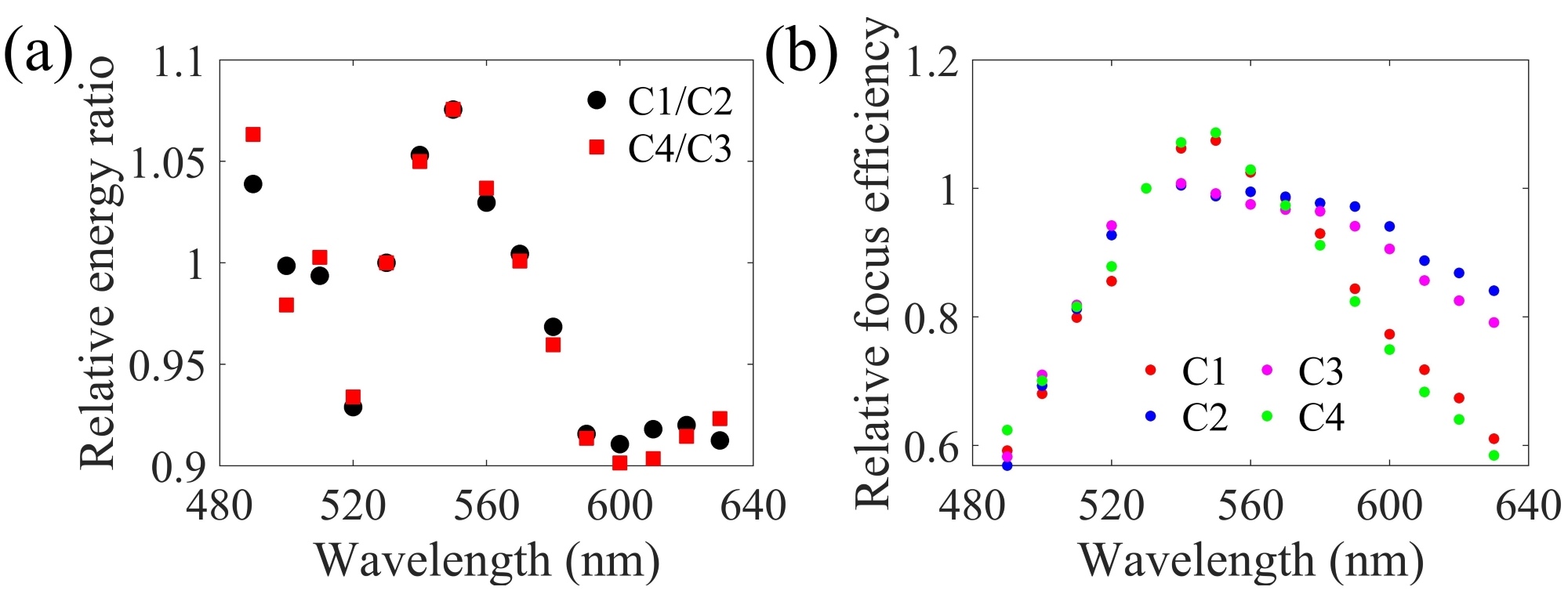


**Figure S15.** The relative energy ratios and focusing efficiencies of the four channels.

**Note S5: Effect of misalignment tolerance and structural dimensional error on the performance of bilayer metasurfaces**

Here, we consider the effect of the misalignment tolerance $\delta$ between the top and bottom metasurfaces (Figure S16a) and the transverse structural dimensional error $\Delta$ of the nanopillars (Figure S16b) on the performance of the free-standing bilayer metasurface. The metasurface M9 is chosen as the target of the study, which is used to realize the four-channel phase and energy control, the C1 and C2 polarization channels are considered here. To assess the performance of M9, we employ three metrics: relative FWHM, relative energy ratio of C1 and C2 channels, and relative focusing efficiency. To highlight variation, the metrics at the design wavelength (532 nm) are treated as the unit.

The bottom metasurface position is fixed, and fixing $\delta_{x}$ (or $\delta_{y}$) to 0 and changing $\delta_{y}$ (or $\delta_{x}$). Figure S17 shows the effect of misalignment tolerance on relative FWHM, relative energy ratio, and relative focusing efficiency. The variations caused by misalignment within 30 nm is no more than 5% compared to perfect alignment ($\delta=0$). The electron-beam lithography (EBL) system reported in the Ref. [36] has sub-20 nm alignment accuracy.


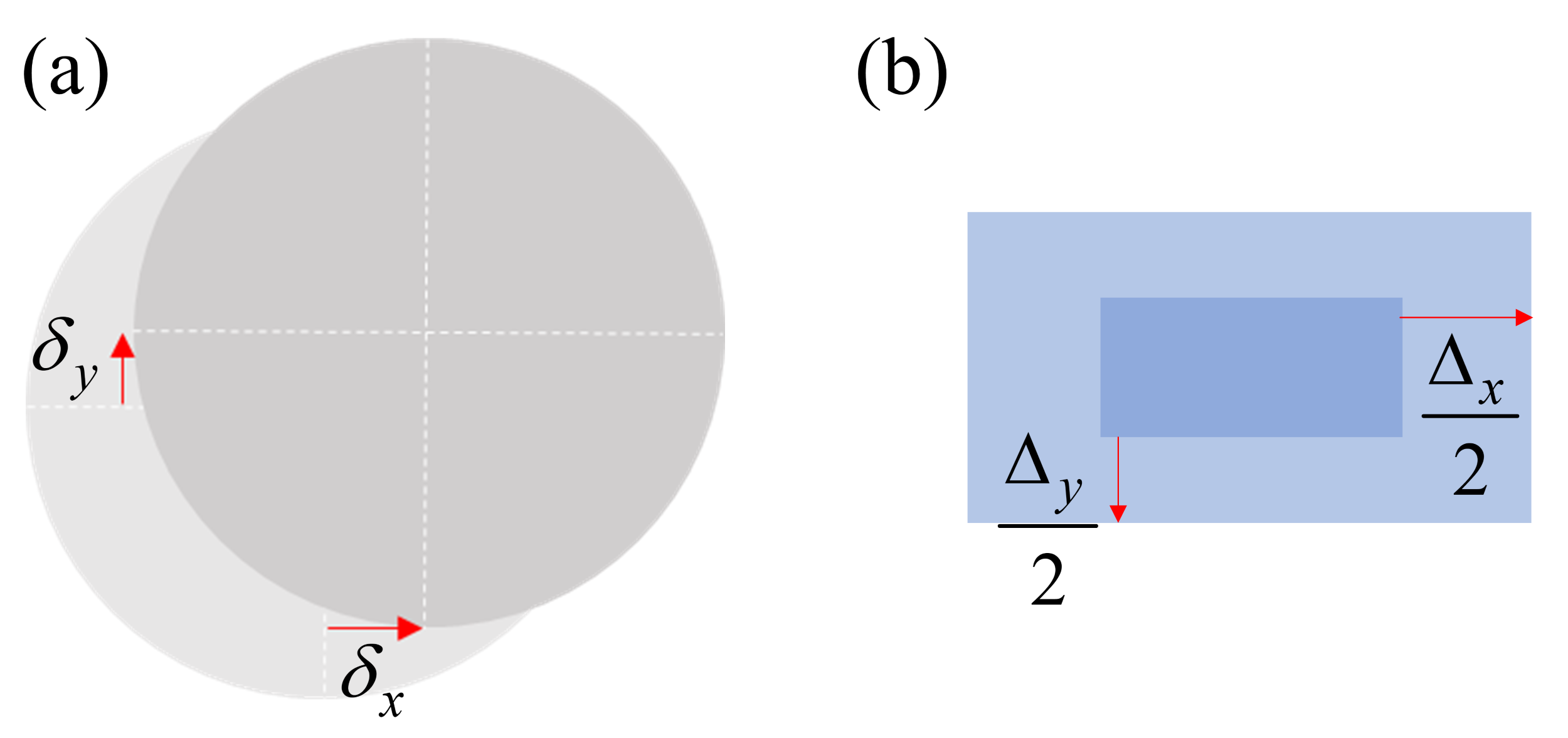


**Figure S16.** (a) Lateral misalignment of the bilayer metasurface (top view). (b) Lateral dimensional error of nanopillar (top view).


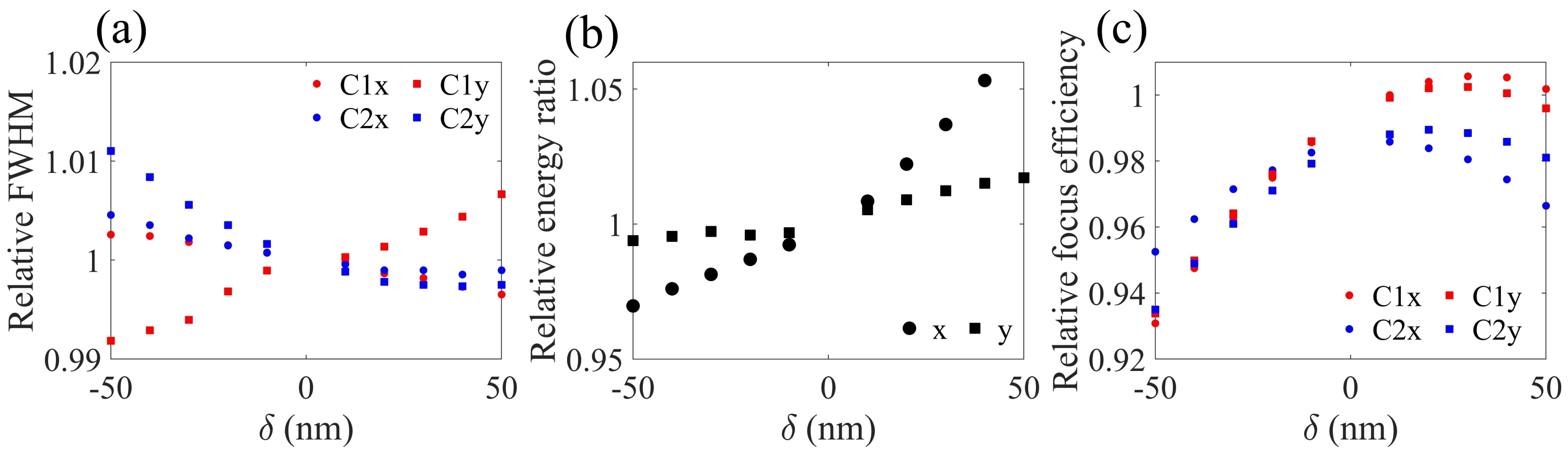


**Figure S17.** Effect of lateral misalignment $\delta$ ($\delta_{x}$ and $\delta_{y}$) on the performance of the metasurface M9.

The fifteen sets of errors generated by the random function within the range of 3, 5, 10, 15, and 20 nm were imposed on the lengths and widths of all nanopillars that make up metasurface M9. The effect of dimensional errors of the nanopillars on the performance of M9 is shown in Figure S18. Figure S18 indicates that the performance variations due to structural dimensional errors within 5 nm do not exceed 5%. In addition, dimensional errors significantly affect the efficiency of metasurfaces, but a lesser effect on FWHM and the energy ratio between the C1 and C2 channels.


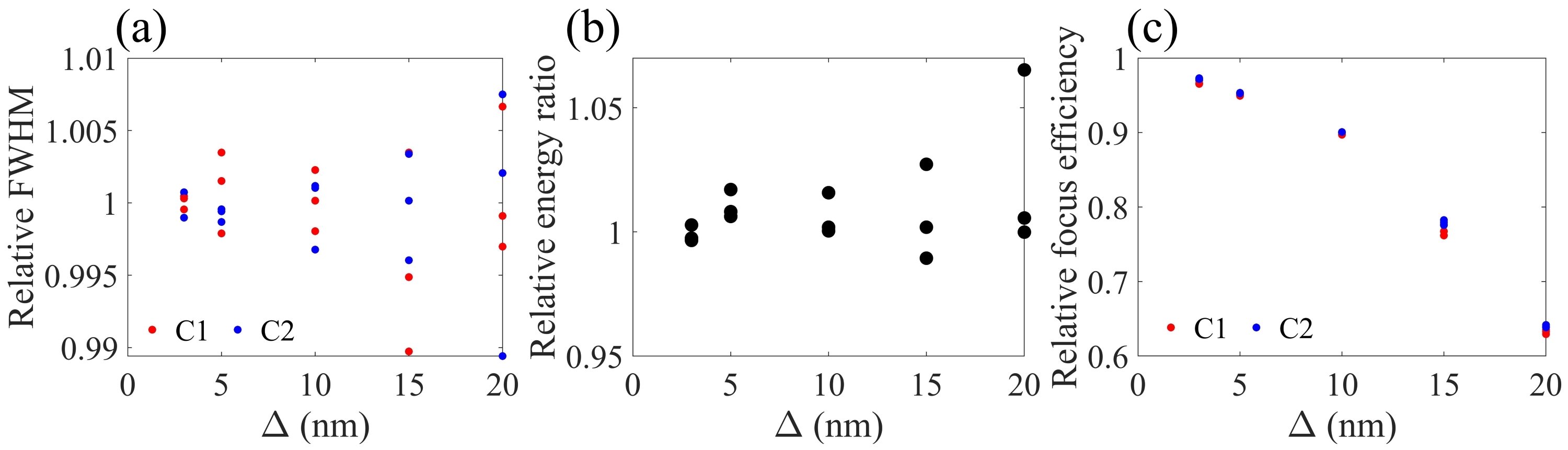


**Figure S18.** Effect of dimensional error (Δ) on the performance of the metasurface M9.

**Note S6: Generating four-channel vortex beams by stacked metasurfaces**

Here, we utilize stacked metasurfaces to generate focused vortex beams, whose function is consistent with M1. The configuration of the meta-atom is shown in Figure S19, where S denotes the distance between the bottom nanopillar and the top nanopillar. It should be noted that the top layer nanopillars function as HWPs, while the bottom layer nanopillars act as quarter-wave plates, which is completely opposite to the meta-atoms of M1. The reason is that the bottom layer nanopillars have a low refractive index contrast, making it difficult to find enough HWP nanopillars under the condition of low height. Therefore, the four-channel complex amplitudes of the stacked metasurfaces can be described by Equation (S16). The length, width, and height of the bottom nanopillars are 310 nm, 145 nm, and 700 nm, respectively, and other parameters are consistent with free-standing bilayer meta-atoms.

The energy ratios, focusing efficiencies, and OAM purities of the four channels as functions of the interlayer spacer thicknesses S between the two layers of nanopillars are shown in Figure S20. Note that these three metrics tend to stabilize as S increases. Additionally, the energy ratio between channels C1 and C2 (or C4 and C3) is not the designed 1:1. This could be due to the coupling and reflection existing between the two layers of nanopillars.^[37]^ Although energy control fails to meet expectation, Figure S20c shows that phase manipulation is successfully achieved, as evidenced by the OAM purities of all four channels being comparable to those of M1.The distributions of focal plane intensity, phase, and OAM purity for S = 0, 0.4, 0.8, and 1.2 μm are presented in Figures S21 and S22, respectively.


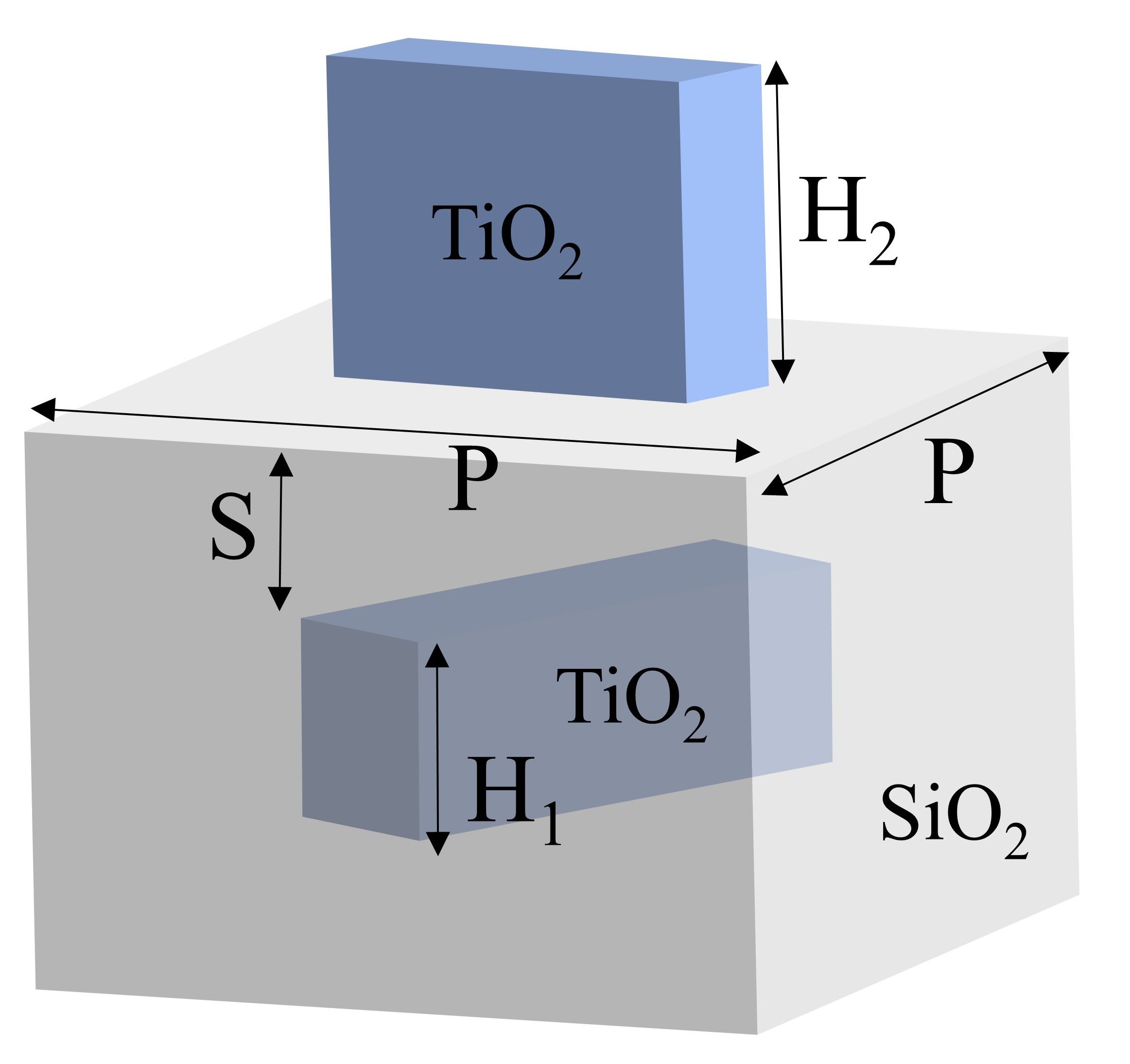


**Figure S19.** Configuration diagram of the meta-atom constituting the stacked metasurfaces.


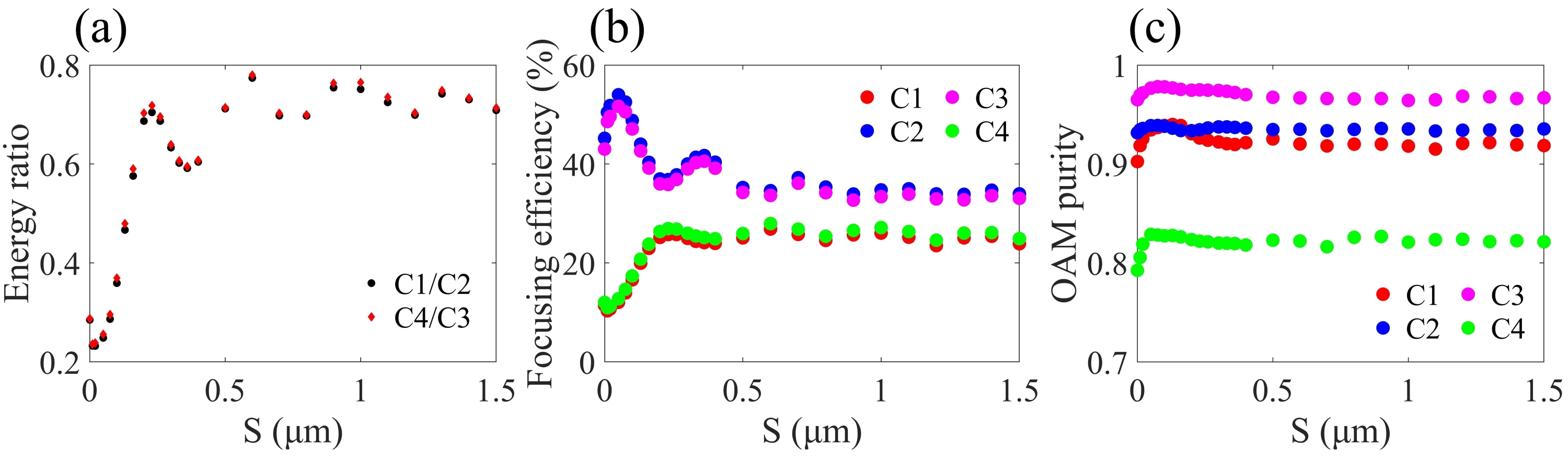


**Figure S20.** The energy ratios, focusing efficiencies, and OAM purities of the four channels as functions of the interlayer spacer thicknesses S.


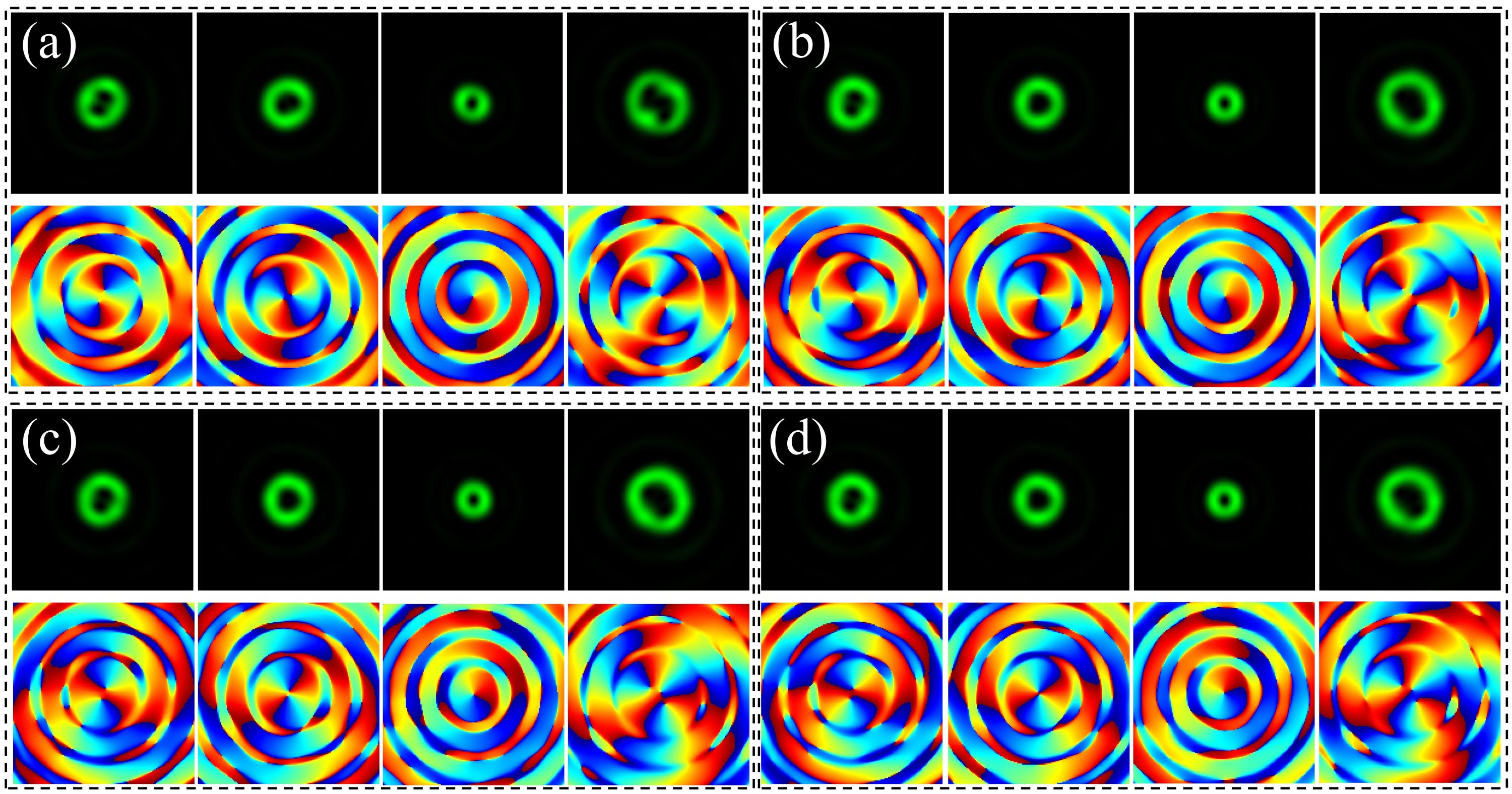


**Figure S21.** The distributions of focal plane intensity and phase for S = 0 (a), 0.4 (b), 0.8 (c), and 1.2 μm (d).


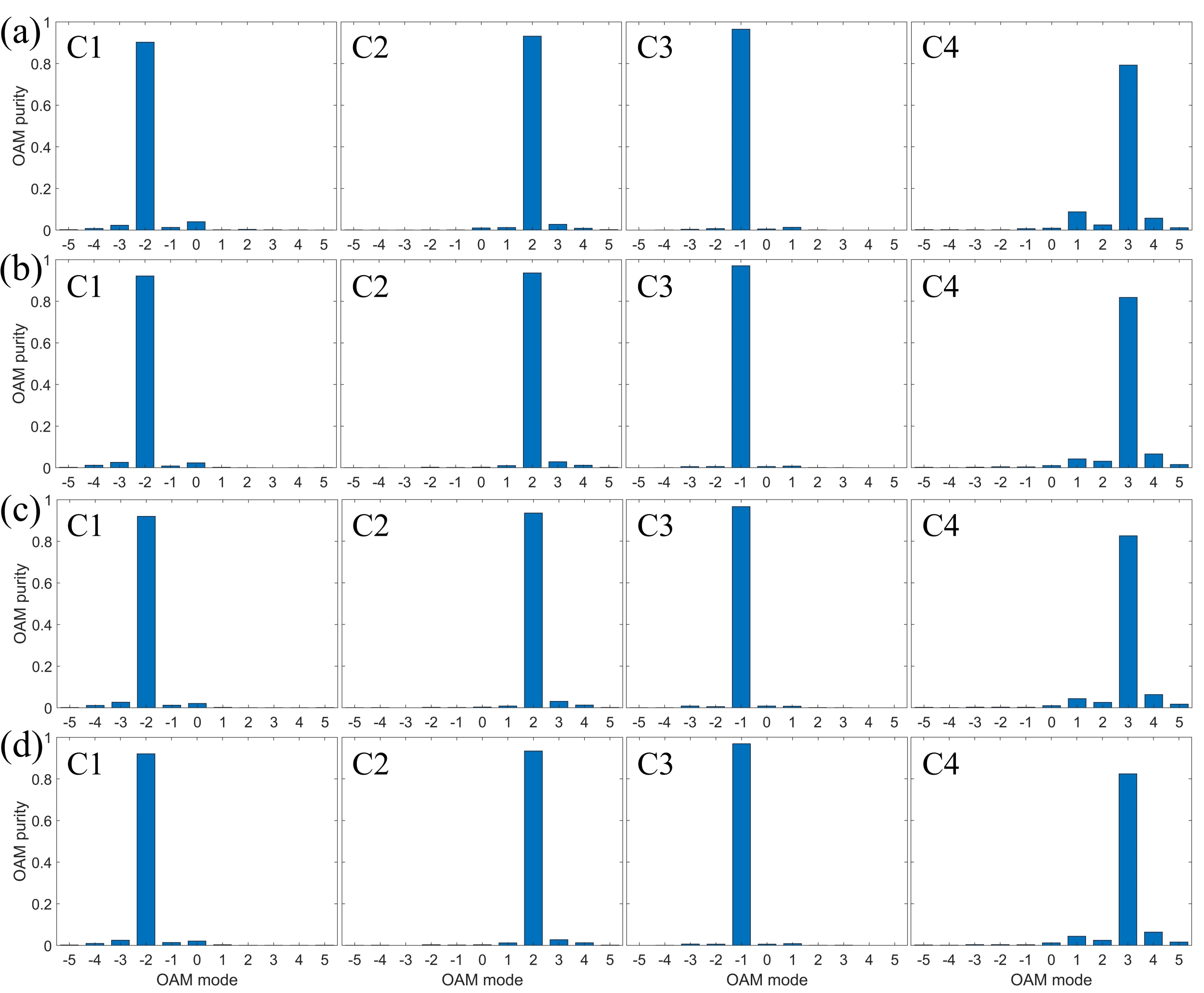


**Figure S22.** The distributions of focal plane intensity, phase, and OAM purity for S = 0 (a), 0.4 (b), 0.8 (c), and 1.2 μm (d).

**References**

1. K. Zhang, Y. Yuan, X. Ding, H. Li, B. Ratni, Q. Wu, J. Liu, S.N. Burokur, J. Tan, Polarization‐Engineered Noninterleaved Metasurface for Integer and Fractional Orbital Angular Momentum Multiplexing, Laser Photonics Rev. 2021, 15, 2000351, <https://doi.org/10.1002/lpor.202000351>.
2. V. Liu, S. Fan, S4: A free electromagnetic solver for layered periodic structures, Comput. Phys. Commun. 2012, 183, 2233, <https://doi.org/10.1016/j.cpc.2012.04.026>.
3. E.D. Palik, Handbook of Optical Constants of Solids I- III, Academic press, 1998.
4. C. Chen, Y. Wang, M. Jiang, J. Wang, J. Guan, B. Zhang, L. Wang, J. Lin, P. Jin, Parallel Polarization Illumination with a Multifocal Axicon Metalens for Improved Polarization Imaging, Nano Lett. 2020, 20, 5428, <https://doi.org/10.1021/acs.nanolett.0c01877>.
5. A. Niv, G. Biener, V. Kleiner, E. Hasman, Manipulation of the Pancharatnam phase in vectorial vortices, Opt. Express 2006, 14, 4208, <https://doi.org/10.1364/oe.14.004208>.
